# Designing In Situ Grown Ternary Oxide/2D Ni-BDC MOF Nanocomposites on Nickel Foam as Efficient Electrocatalysts for Electrochemical Water Splitting

**DOI:** 10.1021/acsmaterialsau.2c00073

**Published:** 2022-12-28

**Authors:** Ebrahim Sadeghi, Naeimeh Sadat Peighambardoust, Sanaz Chamani, Umut Aydemir

**Affiliations:** † Koç University Boron and Advanced Materials Applications and Research Center (KUBAM), Sariyer, Istanbul34450, Turkey; ‡ Graduate School of Sciences and Engineering, Koç University, Sariyer, Istanbul34450, Turkey; § Department of Chemistry, Koç University, Sariyer, Istanbul34450, Turkey

**Keywords:** electrocatalysis, overall water splitting, metal−organic framework, metal oxides, nanocomposites

## Abstract

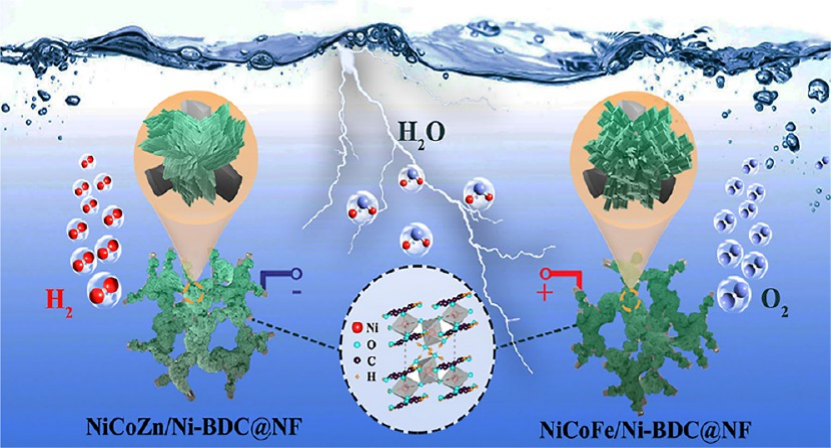

The security of future energy, hydrogen, is subject to designing high-performance, stable, and low-cost electrocatalysts for hydrogen and oxygen evolution reactions (HERs and OERs), for the realization of efficient overall water splitting. Two-dimensional (2D) metal–organic frameworks (MOFs) introduce a large family of materials with versatile chemical and structural features for a variety of applications, such as supercapacitors, gas storage, and water splitting. Herein, a series of nanocomposites based on NCM/Ni-BDC@NF (N=Ni, C=Co, M:F=Fe, C=Cu, and Z=Zn, BDC: benzene dicarboxylic acid, NF: nickel foam) were directly developed on NF using a facile yet scalable solvothermal method. After coupling, the electronic structure of metallic atoms was well-modulated. Based on the XPS results, for the NCF/Ni-BDC, cationic atoms shifted to higher oxidation states, favorable for the OER. Conversely, for the NCZ/Ni-BDC and NCC/Ni-BDC nanocomposites, cationic atoms shifted to lower oxidation states, advantageous for the HER. The as-prepared NCF/Ni-BDC demonstrated prominent OER performance, requiring only 1.35 and 1.68 V versus a reversible hydrogen electrode to afford 10 and 50 mA cm^–2^ current densities, respectively. On the cathodic side, NCZ/Ni-BDC exhibited the best HER activity with an overpotential of 170 and 350 mV to generate 10 and 50 mA cm^–2^, respectively, under 1.0 M KOH medium. In a two-electrode alkaline electrolyzer, the assembled NCZ/Ni-BDC (cathode) ∥ NCF/Ni-BDC (anode) couple demanded a cell voltage of only 1.58 V to produce 10 mA cm^–2^. The stability of NCF/Ni-BDC toward OER was also exemplary, experiencing a continuous operation at 10, 20, and 50 mA cm^–2^ for nearly 45 h. Surprisingly, the overpotential after OER stability at 50 mA cm^–2^ dropped drastically from 450 to 200 mV. Finally, the faradaic efficiencies for the overall water splitting revealed the respective values of 100 and 85% for the H_2_ and O_2_ production at a constant current density of 20 mA cm^–2^.

## 1 Introduction

The inevitably rapid depletion of fossil fuels, progressively rising energy demands, and the escalation of environmental concerns with the use of carbon-bearing fuels are the driving forces that have begotten intense efforts to secure alternative energy sources which are abundant, viable, green, and carbon dioxide (CO_2_)-free.^1−3^ Hydrogen (H_2_) with high energy density, environmental friendliness, and excellent energy conversion efficiency is globally accepted as a potential substitute for carbon-containing fuels.^3,4^ Among the multiple proposed and investigated approaches to produce H_2_, the electrochemical dissociation of water—powered by renewable energy resources (e.g., wind and solar) into oxygen (O_2_) and H_2_—stands out owing to its low-cost, high-purity H_2_ production, zero CO_2_ emission, and no need for high temperature.^5−8^ The two key half-reactions of hydrogen evolution (HER), that is, 2H_2_O + 2e^–^ → H_2_ + 2OH^–^ and oxygen evolution (OER), that is, 4OH^–^ → O_2_ + 2H_2_O + 4e^–^ occur, respectively, at the cathodic and anodic sides under alkaline medium and in total require a minimum potential of 1.23 V under ambient conditions.^4,9^ The conversion efficiency of overall water splitting strongly relies on the merits of implemented electrocatalysts to minimize the activation energy and drive the electrolysis forward.^10−12^

The relentless pursuit to replace highly active but not stable noble metal-based (Pt/C for the HER and RuO_2_/IrO_2_ for the OER) electrocatalysts has created a surge of research on the design and development of materials involving earth-abundant elements with which not only high-performance can be expected but also durability can be perceived.^1^ So far, a miscellaneous series of transition metal (TM:Fe, Co, Ni, Mn, Cu, V, and Cr)-based catalysts including but not limited to phosphides, oxides, oxyhydrates, perovskites, nitrides, borides, and sulfides^12−17^ have been architected to simultaneously catalyze both HER and OER. Thanks to their high abundance, multi-valence state, intrinsic activity, and rich redox properties,^14,15,18^ they can rival the noble metal-based catalysts and even surpass them in some cases. Beyond the preceding merits, transition-metal oxides (TMOs) possess some additional values such as sufficient tolerance against corrosion within a wide range of electrochemical window in alkaline solutions,^2^ containing abundant structural defects^19^ which make them excellent candidates for the OER, HER, and overall water splitting. Moreover, it has been reported that binary/ternary TMOs could promote the kinetic of water splitting, ascribing to the peculiar morphology grown on the surface for facilitated ion transfer,^20^ creating distinctive crystal/crystal interfaces for better adsorption/desorption of intermediates,^21^ improved electronic conductivity,^22,23^ and the effect of synergy between multiple metal centers.^23^

Despite the numerous advantages of 3d TM-derivative materials, their performance under strong basic/acidic conditions and high current densities is limited^18^ and thus not practical for real applications. To overcome this impediment, the construction of a composite with other materials such as metal–organic frameworks (MOFs) with inherent mechanical stability could be a promising solution.^24−26^ MOFs, a newly emerged family of crystalline coordination polymers composed of metal ions (e.g., Fe, Co, Ni, and Cu) attached to organic ligands with tunable chemical composition, high electrochemical surface area (ECSA), hollow structures, and controllable morphology,^13,18^ have gained fame for a variety of applications such as electrocatalytic water splitting,^27−29^ gas storage,^30,31^ chemical sensing,^32,33^ supercapacitors,^34,35^ and metal-air batteries.^36−38^ Regarding catalysis, the unstable coordinated solvent molecules in the MOF structure can easily detach in the course of the reaction and form coordinatively unsaturated metal centers (CUMCs).^39^ Depending on coordinated metal components, the CUMCs may function as typical Lewis acid centers to demonstrate versatile catalytic activities. However, the poor electrical conductivities of MOFs (about 10^–10^ S m^–1^)^40,41^ hamper their implementation in practice as efficient electrocatalysts for the OER and HER.^42−44^

To address the above-stated issue, one approach is to load electrocatalytic MOFs on conductive substrates such as nickel foam (NF). The three-dimensional (3D) open porous framework of NF with a large specific surface area and zigzag channels can further strengthen the electron and mass transport of in situ grown MOFs on the NF backbone.^1^ In fact, self-supported MOF electrocatalysts induce superior electrochemical performances compared to the powder-based systems with the binders such as Nafion,^13,45,46^ which can be attributed to the synergistic effect between MOF-based composites and conductive host.^47^ Additionally, the idea of binder-free hydrothermal synthesis of both ternary mixed TMOs and MOFs using NF as a substrate is interesting in that NF can function as a current collector for direct growth. Moreover, this strategy can minimize binder costs, reduce preparation time, boost ion transport, and strengthen the contact between electrocapacitive materials and substrates. Furthermore, the 3D-order hierarchical nanostructure of MOF/ternary mixed TMOs induced remarkable structural stability and improved electron/ion mobility that are advantageous to obtain superior electrochemical features.^48^ Not to mention that, so far, binary oxides of Ni–Co have been extensively studied for the electrolysis of water. It should be noted that the addition of a third component (oxides of Fe, Cu, and Zn) could in principle demonstrate higher electrochemical performance, owing largely to the enriched oxidation states and synergistic effects of the multi-metal components in the electrodes.^49^

In addition to the abovementioned factors, the synergistic effect between host–guest type composite is another effective parameter to promote the performance of multicomponent composites in oxide electrocatalysts due to their inherently low electrical conductivities.^50^ The strong adhesion of nanocomposite materials (guest) on a porous substrate like NF (host) can enlarge the electroactive surface area and boost the electron transfer within the structure. Keeping these in mind, we herein focus on the architecture of an entirely novel nanocomposite based on Ni–Co–M oxide (M = Fe, Cu, and Zn)/Ni-BDC MOF@NF (see Figure S1 as an instance) for highly efficient OER, HER, and overall water splitting. For the construction of the electrocatalysts, a simple two-step hydrothermal method was adopted. With this design, multiple synergistic effects between NF substrate, ternary Ni–Co–M oxide nanocomposite, and Ni-BDC MOF [Ni_3_(OH)_2_(tp)_2_(H_2_O)_4_] (tp = C_8_H_4_O_4_^2–^)^51,52^ could be presumed. From the electrochemical experiments under an alkaline medium, Ni–Co–Fe oxide/Ni-BDC@NF generated a current density of 10 mA cm^–2^ at an ultra-low overpotential (η) of 120 mV toward OER, Ni–Co–Zn oxide/Ni-BDC@NF afforded the 10 mA cm^–2^ at a small overpotential of 170 mV toward HER, and finally, once these two best OER and HER catalysts were combined to drive overall water splitting, the couple demanded only a cell voltage of 1.58 V to deliver 10 mA cm^–2^.

## 2 Results and Discussion

### 2.1 Chemical Studies

**Figure 1:**
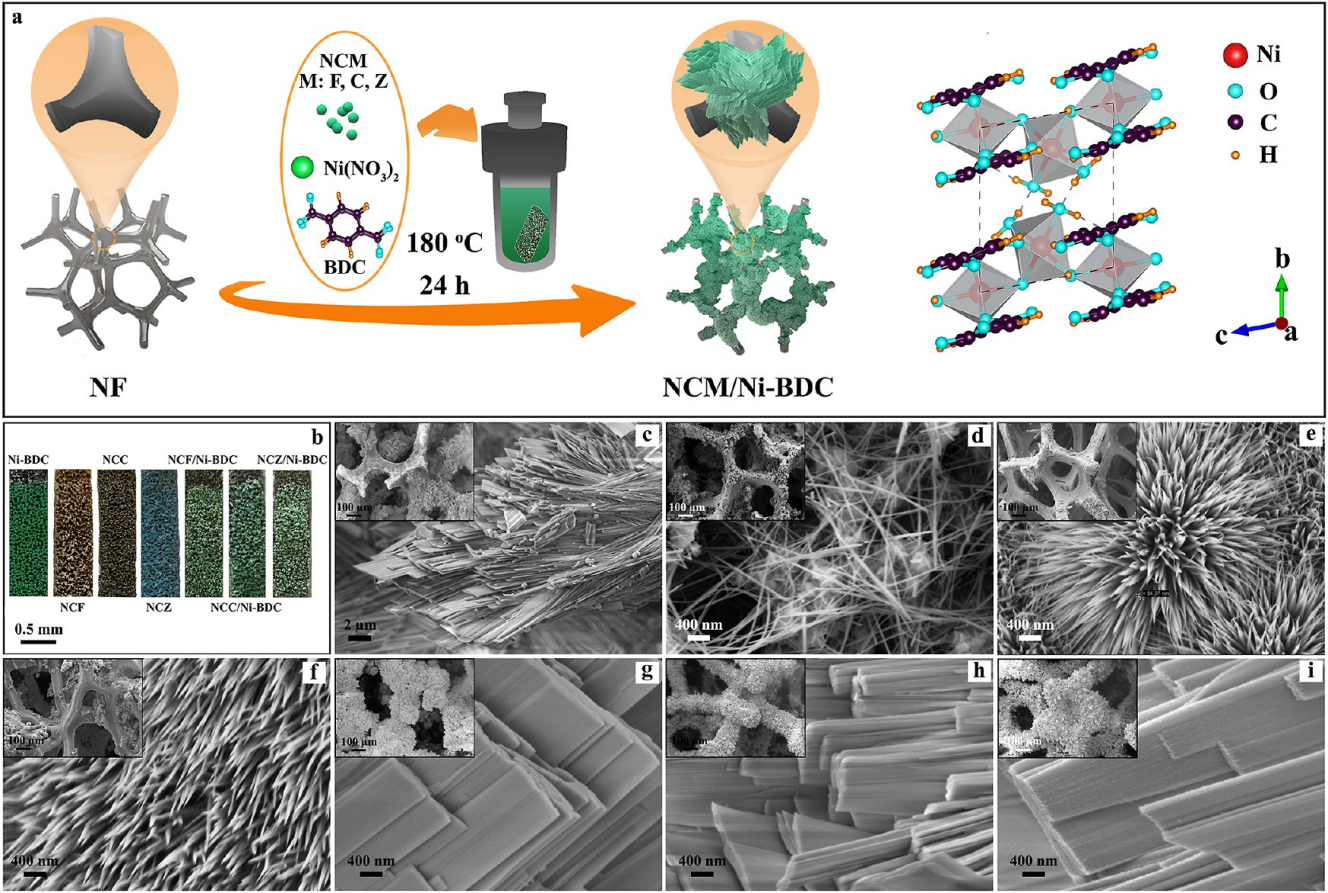
(a) Schematic illustration of nanocomposite catalyst fabrication, NCM/Ni-BDC @NF (N=Ni, C=Co, M:F=Fe, C=Cu, and Z=Zn, BDC: benzene dicarboxylic acid, NF: nickel foam), (b) digital photographs of various in situ grown catalysts on NF with distinctive colors, (c–i) SEM images of Ni-BDC, NCF, NCC, NCZ, NCF/Ni-BDC, NCC/Ni-BDC, and NCZ/Ni-BDC, respectively (inset: low magnification images show the growth of the catalyst on the NF skeleton).

The synthetic procedures to produce ternary oxides and nanocomposites are thoroughly described in the Experimental Section. The schematic image (Figure 1a) manifests the two-step hydrothermal process to directly grow the nanocomposites of NCM/Ni-BDC@NF (N=Ni, C=Co, M:F=Fe, C=Cu, and Z=Zn, BDC: benzene dicarboxylic acid, NF: nickel foam). The colorful growth of bare Ni-BDC, ternary oxide NCM, and NCM/Ni-BDC nanocomposite on NF indicates the successful coverage of all investigated materials on the substrate (Figure 1b). Scanning electron microscopy (SEM) was employed to better understand the morphology of materials grown on NF. From the high-magnification photograph in Figure 1c, the heavily stacked lamellar sheets of Ni-BDC can be visualized which is in line with previous reports.^14,53^ The inset image shows the coverage of Ni-BDC on the 3D macroscopic porous backbone of the NF substrate, the pore width of which can reach several hundreds of micrometers.

Figure 1d–f depicts the microstructure of NCM@NF. For these materials, urea is a crucial ingredient in a typical hydrothermal recipe. Following the mixing of the starting materials and once the metal ions including Ni^2+^, Co^2+^, and M^*n*+^ (M^*n*+^ = Fe^3+^, Cu^2+^, and Zn^2+^) absorb on the NF surface, urea slowly decomposes into hydroxyl and carbonate anions to adjust pH. In this sense, metal ions are involved in the reaction with anions and produce the Ni–Co–M precursor. With the reaction going forward, the concentration of anions declines in the reaction which brings about an array of nanowire morphology for Ni–Co–Fe^54^ and urchin-like morphology for Ni–Co–Cu and Ni–Co–Zn,^55,56^ which can be seen, respectively, in Figure 1d–f. The diameter of the nanowires was estimated to be 20–60 nm, while the diameter of nanoneedles of urchin structures was in the range of 40–90 nm. In addition, the inset images reveal the full coverage of the NF skeleton with a uniform thin layer of ternary oxide. From the low-magnified micrographs (inset of Figures 1d–f and S2), the NF-templated 3D nanostructure of metal oxides can furnish a 3D interconnected pathway for the facilitated mass and electron transfer during faradaic redox reactions.^57^ It must be noted that previous studies calcined the materials on NF; nonetheless, in this work, except for Ni–Co–Zn oxide@NF (NCZ), we avoided the calcination step for the sake of electrochemical examinations.

The topographies of NCM/Ni-BDC@NF are portrayed in Figure 1g–i. It is evident that the closely packed lamellar morphology is maintained after the addition of ternary oxide into the 3D structure of Ni-BDC. A comparison of inset images of Ni-BDC@NF and NCM@NF with NCM/Ni-BDC@NF suggests that the coverage of NCM/Ni-BDC nanocomposites on NF is relatively thicker. Besides, microstructural imaging in Figure S2 represents the growth of ternary metal oxides on the NF backbone at lower magnification, featuring a better view of the metal oxides’ coverage of NF. Likewise, Figure S3 portrays a top-view SEM image of blank Ni-BDC and its nanocomposites, revealing the dense deposition of layers on the NF substrate with poor porosity. What is more, the energy dispersive X-ray spectroscopy (EDS) elemental mappings were performed on a large portion of materials on the NF substrate to show the distribution of all elements throughout the entire substrate (see Figures S4 and S5). Eventually, the X-ray fluorescence spectrometry (XRF) quantified the concentrations of TMs within the structure of NCM, and the results are tabulated in Table S1. The presence of excess Ni compared to other elements is not surprising, demonstrating the Ni extraction from NF under heat and pressure during the hydrothermal procedure.

Figure 1a illustrates the crystal structure of Ni-BDC along the *a*-axis, residing in the triclinic system (space group *P̅*1).^58^ The material demonstrates a layered arrangement perpendicular to the *b*-axis and parallel to the ac plane. Each layer consists of 1D corner sharing Ni(II)O_6_ octahedra chains elongated along the *c*-axis and joined to one another through bridging bidentate tp = C_8_H_4_O_4_^2–^ anions along the *a*-axis. Layers are connected by hydrogen bonds between Ni-coordinated water molecules with which the integrity of the structure is sustained. In this structure, the most intense plane (100) provides the most exposed structure for the electron transfer and diffusion of electrolyte solution. The 1D Ni(II)O_6_ octahedra chains along the *c*-axis create a conductive route for electrons, and at the same time, the layers parallel to the ac plane—with interspaces between each layer—facilitate the storage and diffusion of electrolyte solution. Such a layered and sophisticated structure can be beneficial for electrochemical applications.^51^

X-ray diffraction (XRD) is an excellent tool to examine the chemical composition and crystallographic structure of the as-obtained materials. The XRD patterns of ternary oxide NCM—scratched off from NF—can be visualized in Figure S6. As demonstrated in this figure, the XRD peaks of NCF can be constructed from Fe_2_O_3_ (Card no.: 72-0469) [at 2-theta of 23.03, 32.86, and 54.08° corresponding to (012), (104), and (116)], spinel ferrite, Fe_3_O_4_ (Card no.: 74-0748) [at 2-theta of 30.33, 35.64, 57.34, and 62.89° relating to (220), (311), (511), and (440)], Co_2_(OH)_2_CO_3_ [at 2-theta of 14.60, 17.44, 24.12, 27.68, 34.65, 32.80, 36.56, 39.65, 42.06, 43.41, 48.96, 51.86, 59.74, and 61.04° pertaining to (020), (120), (220), (001), (121), (−211), (330), (221), (250), (−241), (260), (401), (−212), and (370), refer to,^59^ and Ni(OH)_2_ (Card no.: 38-0715) [at 2-theta of 11.34, 23.03, 38.78, and 46.03° indexing to (003), (006), (015), and (018)] phases. In the case of NCC, the possible phases in the sample were identified to be CuO (Card no.: 73-6023) [at 2-theta of 35.43, 38.60, and 49.39° assigning to (−111), (111), and (−202)], Cu(OH)_2_ (Card no.: 72-0140) [at 2-theta of 33.86, 39.71, 53.90, 56.17, and 62.60° belonging to (002), (130), (132), (151), and (200)], and Cu_2_(OH)_2_CO_3_ (Card no.: 72-0075) [at 2-theta of 14.65, 17.46, 24.11, 28.02, 29.65, 30.70, 36.56, 42.14, 44.98, 47.42, 50.93, and 59.92° appertaining to (020), (120), (220), (011), (310), (−201), (330), (250), (−411), (−151), (520), and (−361)]. It is important to mention that Co_2_(OH)_2_CO_3_ and Cu_2_(OH)_2_CO_3_ are isostructural, and both may contain some Cu or Co species in their structures which cannot be easily distinguished by the XRD patterns. Interestingly, Co_2_/Cu_2_(OH)_2_CO_3_ has a typical nanowire-like structure, and according to several reports, this structure encompasses a large number of active sites for an electrocatalytic reaction.^60−62^ Regarding the NCZ, the reflections were found to be consisting of spinel-type ZnCo_2_O_4_ (Card no.: 73-1702) [at 2-theta of 36.40, 44.50, and 59.74° correlating with (331), (400), and (511)], NiOOH (Card no.: 9012319) [at 2-theta of 12.95° and 24.98° connecting with (111) and (222)], and CoCO_3_ (Card no.: 78-0209) [at 2-theta of 33.28, 38.62, 42.84. 46.77, 53.76, and 62.16° corresponding to (104), (110), (113), (202), (116), and (122)]. Finally, it is of high significance to note that we also detected metallic Zn via XRD at 2-theta angles of around 36.32, 39.15, and 43.25°.

It is not surprising to receive multiple oxides and hydroxides prior to calcination. Besides, there might be a very minor peak shift in the peak positions of ternary mixed oxides which can be attributed to the partial substitution of the elements in their structures. As mentioned above, calcination removes nearly all −OH^–^ and −CO_3_^2–^ species, conducing the formation of ternary oxide which in terms of the crystal structure resembles that of cubic Co_3_O_4_.^54−56,63^ For this, we likewise examined the XRD pattern of a representative sample after calcination at 350 °C for 5 h. The XRD pattern of this sample is identical to the reported Co_3_O_4_ phase (see Figure S7a). However, as mentioned earlier, the materials’ preparation in this work does not include the calcination step.

**Figure 2:**
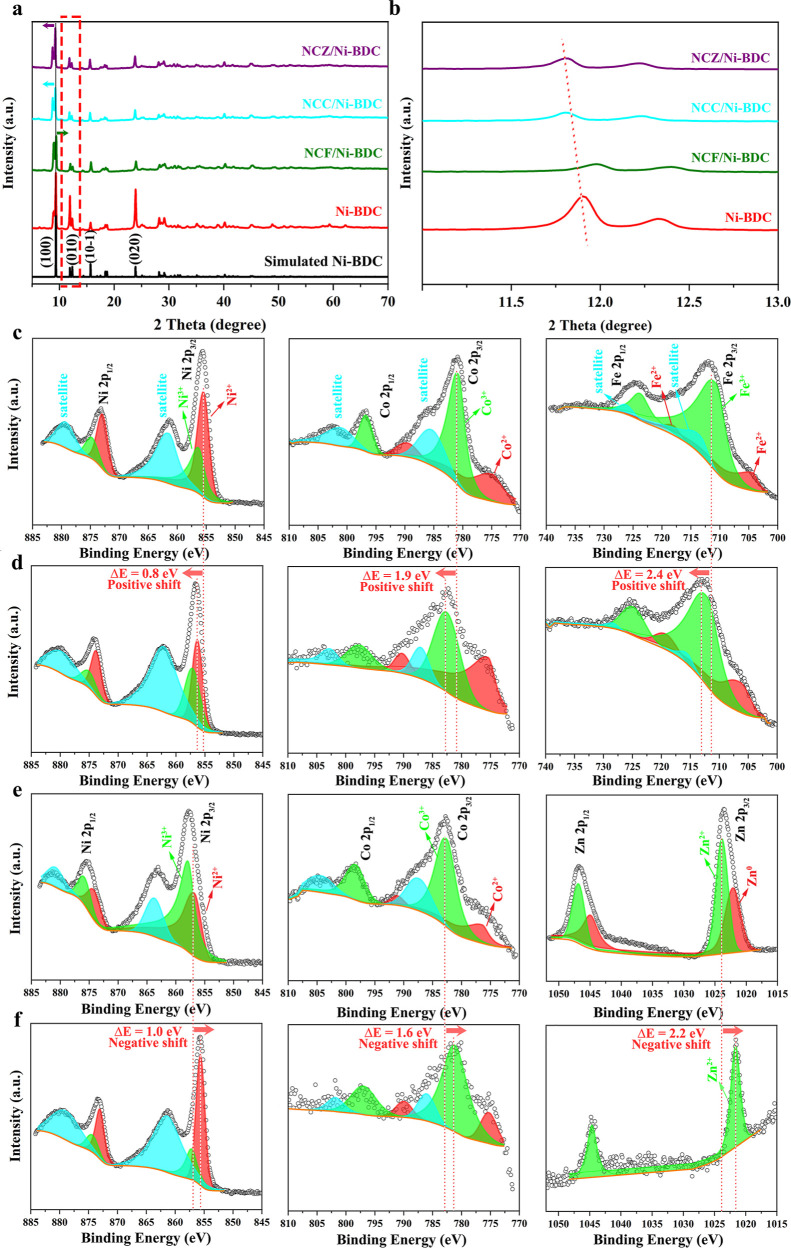
Spectroscopic characterizations of the selected samples. (a) XRD patterns of blank Ni-BDC, NCF/Ni-BDC, NCC/Ni-BDC, and NCZ/Ni-BDC nanocomposites stripped off from NF, (b) shift of XRD (010) peaks for NCF/Ni-BDC, NCC/Ni-BDC, and NCZ/Ni-BDC nanocomposites compared to simulated Ni-BDC pattern, (c,d) XPS Ni 2p, Co 2p, and Fe 2p spectra for NCF and NCF/Ni-BDC, respectively, and (e,f) XPS Ni 2p, Co 2p, and Zn 2p spectra for NCZ and NCZ/Ni-BDC, respectively.

In addition, the XRD patterns of pure Ni-BDC and NCM/Ni-BDC nanocomposites displayed all Bragg reflections related to Ni-BDC, validated by earlier reports (see Figure 2a).^14,51,53^ The coupling of NCM with Ni-BDC resulted in a slight shift in peak positions that we can observe in Figure 2b. The interaction mechanism of MOF ligand and Ni(OH)_2_ was discussed by Zhu et al.^64^ which can be generalized for other phases (refer to the Experimental Section). Interestingly, the (010) for NCF/Ni-BDC shifted to higher 2-theta angles, while for NCC/Ni-BDC and NCZ/Ni-BDC shifted to lower 2-theta angles. This might be explained by the comparison of ionic radii of host metal atoms: Ni^2+^ (69 pm), with heteroatoms, Co^2+^ (74.5 pm), Fe^3+^ (64.5 pm), Cu^2+^ (73 pm), and Zn^2+^ (74 pm). It can be presumed that partial substitution of Ni^2+^ by Fe^3+^ with smaller atomic radius and Ni^2+^ by Cu^2+^/Zn^2+^ with larger atomic radii in the structure of their relevant nanocomposites has induced the modification on the lattice constants of Ni-BDC (host) according to Bragg’s law.^65^ In the meantime, the XRD patterns of NCZ′ and its corresponding nanocomposite, NCZ’/Ni-BDC, can be seen in Figure S7b.

The surface chemical composition and electronic structure of Ni-BDC and Ni-BDC-derived nanocomposites were determined through X-ray photoelectron spectroscopy (XPS). The survey spectra verified the appearance of constituent elements in each sample which is to a great extent consistent with EDS analysis. For blank Ni-BDC (Figure S8), the high-resolution XPS spectrum of C 1s was deconvoluted into three peaks located at binding energies (BEs) of 284.5, 285, and 288.2 eV, which can, respectively, be indexed to C=C (sp^2^), C–C (sp^3^), and carboxylate carbon (O=C–OH) bonds, signifying the presence of terephthalic acid.^66−69^ The core-level Ni 2p XPS spectrum can be divided into two doublet components, Ni 2p_3/2_ and Ni 2p_1/2_ each containing a shake-up satellite peak. For this, two peaks at 855.6 and 872.9 eV are related to Ni^2+^and the other pair at 856.6 and 874.9 eV are correlated to Ni^3+^.^14^

According to the literature, Ni^2+^ can be originated from Ni(OH)_2_^53^ while Ni^3+^ can be stemmed from the octahedral coordination of Ni atoms with terephthalates in the Ni-BDC 3D structure.^70^ The satellite peaks emerged at 861.7 and 879.5 eV,^71,72^ manifesting the existence of nickel–oxygen species.^73^ To explore the presence of surface oxygen content in pure Ni-BDC, the O 1s spectrum was analyzed. The appearance of three peaks at BEs of 531.2, 532.2, and 533.08 eV was respective signals of Ni–O, O–C=O, and absorbed water species (refer to Figure S8).^74^ Concerning NCF/Ni-BDC, first we consider NCF in which Ni 2p, Co 2p, and Fe 2p spectra have been separated into two doublet peaks, one at lower BE (2p_3/2_) and the other at higher BE (2p_1/2_) (see Figure 2c). The spin–orbit splitting values of Ni 2p (Δ_Ni_), Co 2p (Δ_Co_), and Fe 2p (Δ_Fe_) were roughly 17, 15, and 13 eV, confirming the coexistence of both oxidation states of 2+ and 3+.^75−77^ Comparison of NCF/Ni-BDC (Figure 2d) with NCF reveals that the Ni 2p, Co 2p, and Fe 2p peaks in NCF/Ni-BDC shifted to higher BEs, demonstrating the lower electron density of Ni, Co, and Fe atoms in NCF/Ni-BDC and thereby stronger electronic interactions between NCF and Ni-BDC. For the O 1s in NCF, three peaks centered at 529.4, 531.08, and 531.9 eV correspond to M–O bonds,^55,63^ defective sites with low oxygen coordination (oxygen deficiency),^15,78^ and absorbed water molecules,^68^ separately (see Figure S9a). Furthermore, the analyzed XPS survey, C 1s, and O 1s spectra for NCF/Ni-BDC are portrayed in Figure S9b.

To fit the suitable bonds to the appeared spectra of NCZ/Ni-BDC, initially, the peak fitting of NCZ is discussed. For this, Figure 2e shows that Ni 2p and Co 2p spectra are marked with two oxidation states of 2+ and 3+. However, for the core-level Zn 2p XPS spectrum, we observed four peaks: lower BEs (1022.08 and 1023.9 eV) and higher BEs (1045 and 1046.9 eV) with Δ_Zn_ of approximately 23 eV pertaining to metallic Zn (Zn^0^) and Zn^2+^.^79^ From this, we can infer the presence of Zn^2+^ in ZnCo_2_O_4_ which was also verified by XRD. After coupling NCZ with Ni-BDC, nearly all XPS spectra for the obtained nanocomposite shifted to lower BEs compared to those of NCZ (about 1 eV), reflecting lower oxidation states of Ni, Co, and Zn atoms in NCZ/Ni-BDC (Figure 2f). This finding means that Ni, Co, and Zn possess higher electron densities in NCZ/Ni-BDC, as opposed to NCZ. The XPS survey, C 1s, and O 1s of the above-discussed ternary oxide and corresponding nanocomposite were deconvoluted and are visualized in Figure S9c,d. The O 1s core-level spectrum of NCZ was divided into two peaks, one located at 531.2 eV corresponding to M–O bonds and the other centered at 533.2 eV associated with O–C=O.^80^

Concerning the deconvoluted XPS spectra for Cu 2p in NCC, Figure S10a presents two noticeable doublets which were attributed to Cu 2p_3/2_ and Cu 2p_1/2_ of divalent Cu (II), individually, bearing splitting energy of approximately 19.5 eV.^80^ The lower BEs located at 935.4 and 937.4 eV were coupled with those positioned at higher BEs, 954.9 and 957.3 eV, and were associated with the respective phases of CuO and Cu_2_(OH)_2_CO_3_,^78,81^ in line with XRD results (refer to Figure S6). Moreover, the shake-up satellite peak found at 944.3 eV was meanwhile ascribed to Cu 2p_3/2_. It is worth noting that the Cu 2p spectrum of NCC/Ni-BDC was also analyzed (Figure S10b). The results displayed the movement of peaks to lower BEs with a difference of over 1.0 eV. In addition, the Ni 2p and Co 2p spectra were examined, disclosing similar observations. Analogous to NCZ/Ni-BDC, the NCC/Ni-BDC nanocomposite shifted to lower BEs in comparison with NCC, implying the strong interaction between the two components (NCC and Ni-BDC). As a result of the combination of two components, NCC/Ni-BDC experienced lower oxidation states and higher electron densities. Finally, the O 1s spectrum of NCC exhibited two identical peaks to those of NCZ, positioned at 531.2 and 533.3 eV related to M–O bonds and O–C=O.^80^

More insights into the structural information of the ternary oxide (NCM) and nanocomposites (NCM/Ni-BDC) were acquired via Fourier transform infrared spectroscopy (FT-IR). The left panel of Figure S11 depicts the FT-IR spectra conducted on NCM samples. Starting from the peaks detected at the region 3100–3600 cm^–1^ that were assigned to the O–H stretching vibrations of water molecules present in the samples.^82^ The absorption bands that emerged at 2984 and 2902 cm^–1^ were indexed to C–H stretching vibrations originating from urea. Moreover, the existence of the H–OH functional groups has been witnessed by the appearance of peaks at ∼1512 cm^–1^.^83^ In addition, the bands centered at 1364 and 1044 cm^–1^ corroborated the presence of carbonates.^84^ The respective transmittance peaks located at ∼830 and ∼475 cm^–1^ were associated with Co–O and Ni–O in the framework of ternary oxide.^84−86^ Eventually, the appeared transmittance peaks at 687 cm^–1^ for NCM oxides were correlated with Fe–O, Cu–O, and Zn–O bonds, individually.^83^

The right panel of Figure S11 demonstrates the FT-IR spectra recorded for Ni-BDC and its derivative nanocomposites. The observed peaks at 3608 and 3423 cm^–1^ can be emanated from the stretching vibrations of O–H.^51,87^ The distinctive peaks positioned at 1374 and 1576 cm^–1^ were generated from the symmetric and asymmetric vibrations of −COO^–^ groups, respectively, verifying the presence of the dicarboxylate linker in Ni-BDC.^88^ The division of two modes manifested that the −COO^–^ group of 1,4-BDC is attached to Ni via a bidentate mode.^52^ The absorption bands detected at 1524 cm^–1^ were ascribed to the stretching vibrations of para-aromatic CH groups.^51,52,89^ In addition, the C–H bending vibrations were recognized via the IR bands recorded at the range of 750–880 cm^–1^. Finally, the Ni–O vibration was identified through the bands in the region of 463 and 523 cm^–1^.^89−91^

**Figure 3:**
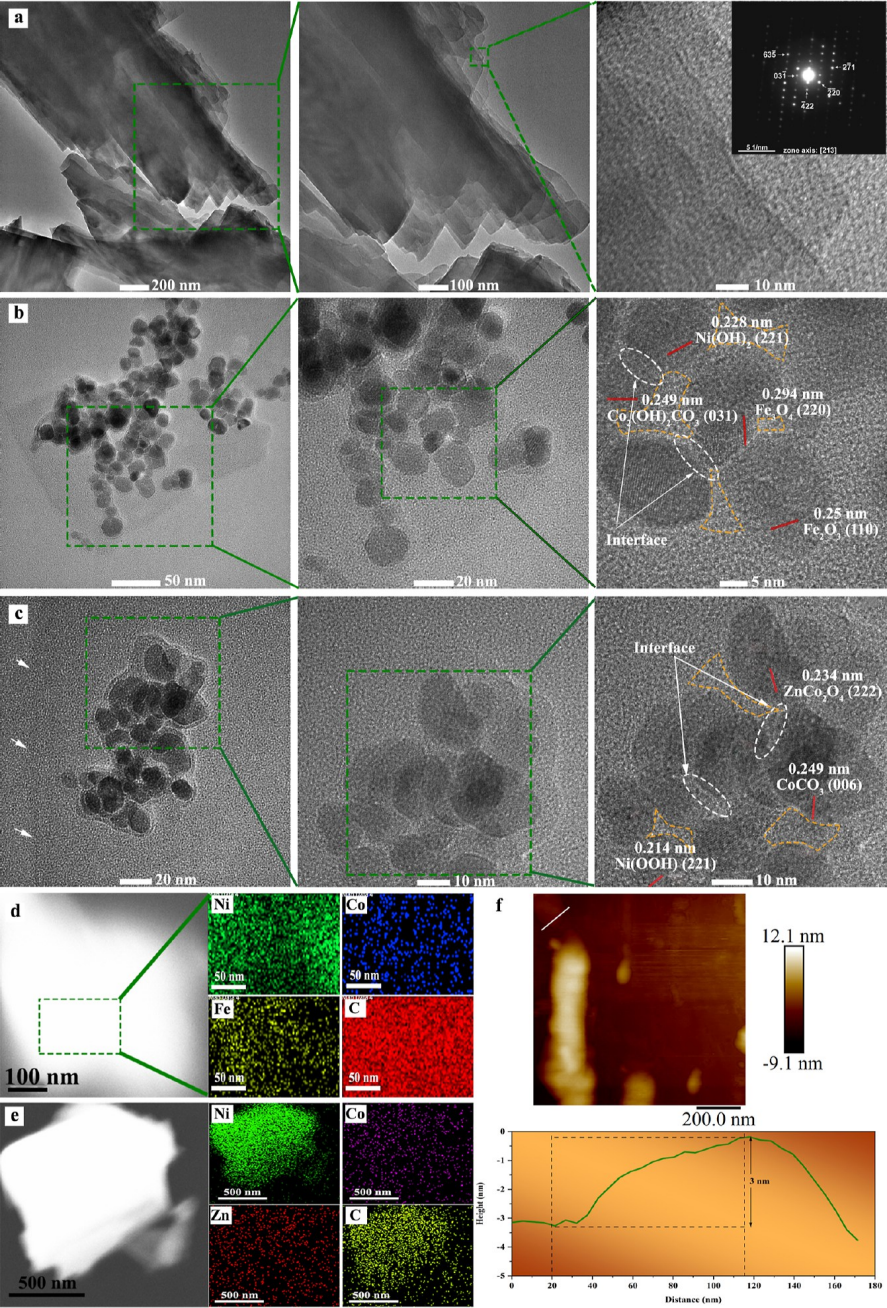
Morphological, structural, and microstructural characterizations of the selected sample. (a) TEM photographs of blank Ni-BDC with different magnifications (inset: SAED image of Ni-BDC), (b,c) TEM and HR-TEM images of NCF/Ni-BDC and NCZ/Ni-BDC, respectively; the first image from left shows the transparent Ni-BDC nanosheet decorated with ternary oxide nanoparticles, third image from left displays the lattice fringes of nanoparticle, white dotted circles show the interfaces, and orange dotted lines show the porosity between nanoparticles, (d,e) HAADF-STEM image and the corresponding STEM-EDS mappings of NCF/Ni-BDC and NCZ/Ni-BDC, respectively, and (f) AFM image and corresponding height profile of blank Ni-BDC.

The microstructure morphology and phase composition of the Ni-BDC and the representative nanocomposites were further scrutinized by transmission electron microscopy (TEM) and high-resolution TEM (HR-TEM) images. To exclude the effect of the NF template, samples were first scratched from the NF and then characterized. From TEM micrographs of pure Ni-BDC (Figure 3a), the 2D nanosheet morphology (the lamellar sheet structure) is further confirmed, as observed by SEM images. Unfortunately, the HR-TEM was not able to resolve the *d*-spacing of Ni-BDC 2D sheets. For this, the selected area electron diffraction (SAED) image of Ni-BDC demonstrated a diffraction pattern with well-defined spots along the [213] zone axis and evidently certified the structure of Ni-BDC (inset image of Figure 3a). From the TEM photographs of NCF/Ni-BDC, the transparency of the Ni-BDC nanosheet under the incident electron beam is discernible on which numerous tiny nanoparticles are deposited (see Figure 3b). We can observe a similar deposition of nanoparticles on nanosheets in the case of NCZ/Ni-BDC nanocomposite (Figure 3c). The polycrystalline structure of nanoparticles on Ni-BDC sheets can be identified by the HR-TEM analysis. It can be claimed that both NCF and NCZ are composed of nanocrystallites <20 nm in size.

The crystalline nanostructures in NCF/Ni-BDC were distinguished by their well-resolved lattice fringes with the interplanar spacings of 0.228, 0.249, 0.294, and 0.25 nm corresponding to the crystalline planes of Ni(OH)_2_ (221), Co_2_(OH)_2_CO_3_ (031), Fe_3_O_4_ (220), and Fe_2_O_3_ (110), individually (Figure 3b: third image from left). Similarly, for the case of NCZ/Ni-BDC, clear crystal planes with a *d*-spacing of 0.234, 0.249, and 0.214 nm were assigned to the presence of ZnCo_2_O_4_ (222), CoCO_3_ (006), and NiOOH (221), separately (Figure 3c: third image from left). In addition, the high porosity of distributed nanoparticles can be substantiated by domains (orange dashed lines) of 5–10 nm in size. From an electrochemical point of view, this distinct mesoporous morphological feature is believed to be beneficial—in that, the electrolyte can reach easily every part of the sample bringing forth rapid ion/electron transfer, thereby leading to enhanced electrochemical reactivity.^92,93^ A typical high-angle annular dark-field scanning transmission electron microscopy (HAADF-STEM) associated EDS elemental mapping (Figure 3d,e) manifested that the obtained NCF/Ni-BDC is composed of Ni, Co, Fe, C, and O elements, and the NCZ/Ni-BDC nanocomposite consists of elements of Ni, Co, Zn, C, and O which for both samples, the elements are uniformly distributed in the representative batch. Finally, atomic force microscopy (AFM) revealed that a single nanosheet of Ni-BDC separated by ultrasonication has a typical thickness of ∼3 nm and a much larger lateral size (Figure 3f), corroborating the two-dimensionality of the Ni-BDC MOF array.

### 2.2 Electrochemical Studies

#### 2.2.1 Electrocatalytic Activity of the Electrodes for the OER and HER

**Figure 4:**
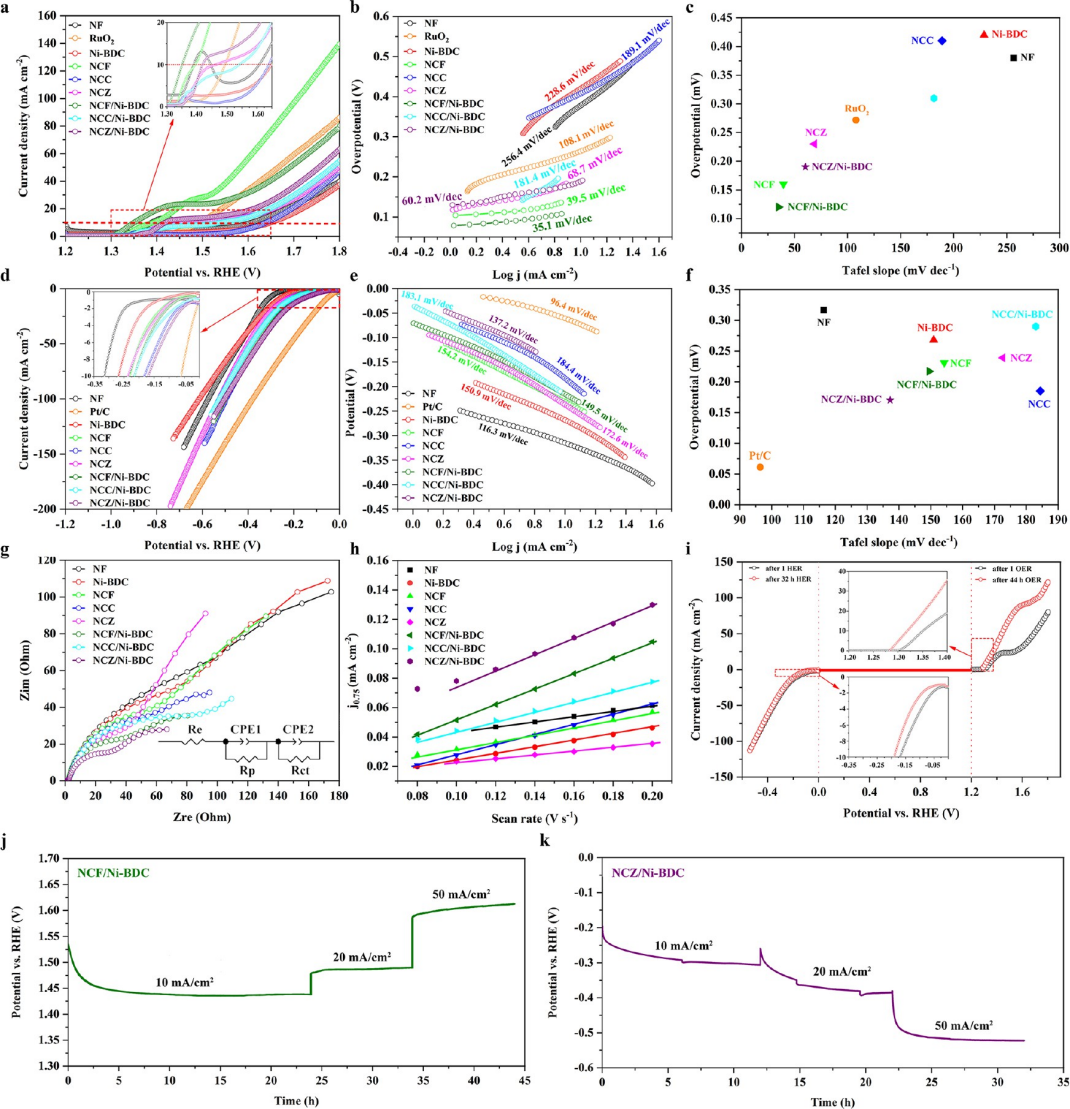
Electrocatalytic oxygen evolution and hydrogen evolution performances. (a,d) OER and HER polarization curves of various catalyst samples: red and blue dotted lines display 10 and 50 mA cm^–2^, respectively, (b,e) OER and HER Tafel plots obtained by polarization curves, respectively, (c,f) comparison of the overpotential at a current density of 10 mA cm^–2^ and Tafel slope on various OER and HER samples, respectively, (g) EIS spectra recorded at 0 V versus RHE, (h) capacitive current density versus scan rate curves for ECSA measurements, (i) OER and HER polarization curves before and after long-term durability measurements for best-performing OER (NCF/Ni-BDC) and HER (NCZ/Ni-BDC) samples, and (j,k) long-term durability tests of NCF/Ni-BDC and NCZ/Ni-BDC for the OER and HER, respectively at 10, 20, and 50 mA cm^–2^.

The electrochemical aspects of the as-prepared catalysts were examined through a series of electrochemical measurements with a standard three-electrode configuration under ambient conditions in 1.0 M KOH solution. Figure 4a depicts the electrocatalytic OER activities of in situ grown materials on the NF substrate along with blank NF and commercial RuO_2_ as a comparison. A small anodic peak was observed below 1.4 V versus reversible hydrogen electrode (RHE) on the polarization curves of some samples, which can be ascribed to the oxidation of Ni^2+^ to Ni^3+^ to form the active intermediate responsible for the OER.^94^ For this, the inset figure demonstrates a better image of the corresponding potential of each catalyst at a 10 mA cm^–2^ current density. Besides, the current density at 50 mA cm^–2^ was also delineated. It should be pointed out that the current densities were normalized to the geometric surface area of the substrate (0.5 cm × 1 cm). From the results, the NCF/Ni-BDC electrode exhibits respective potentials of 1.35 and 1.68 V versus RHE to afford 10 and 50 mA cm^–2^ current densities, respectively (η^10^_OER_ = 120 mV and η^50^_OER_ = 450 mV). We should highlight that sometimes it is very difficult to identify the OER overpotential because of the oxidation of the metal. To deal with this issue, chronopotentiometry at 10 mA cm^–2^ (showing 1.43 V vs RHE) was performed for NCF/Ni-BDC which is discussed later. In addition, NCF delivers the 10 and 50 mA cm^–2^ at η^10^_OER_ = 160 mV and η^50^_OER_ = 350 mV, individually. To compare the results with high-quality electrocatalysts, we have summarized the results of recent reports in Table S2.

It is noteworthy to mention that in the case of the OER, the reaction kinetics—unlike HER—is completely different from one catalyst to another and highly pH-dependent. For instance, the onset overpotential of IrO_2_/RuO_2_ to initiate the reaction is lower compared to that of Ni–Fe; nonetheless, the Tafel slope is smaller for the latter one. This means that while the driving force to start the reaction on IrO_2_/RuO_2_ is lower, the rate of charge transfer/electron transfer is lower than that for the Ni–Fe system in alkaline media. Thus, people have adopted two critical parameters of overpotential at defined current density and Tafel slope to assess the value of electrocatalysts in the OER.^3^

The OER performance of all the as-fabricated samples is summarized in Table S3. By comparing the outcomes, it can be stated that electrodes containing Fe meaningfully outperform other electrodes containing Zn and Cu. Accordingly, from the OER performance point of view for both ternary oxide and nanocomposites, the trend is as follows Fe > Zn > Cu. There is a common property occurring in all materials showing good OER performance which is the presence of spinel oxides in their structures. To be specific and in parallel with XRD results, NCF and NCZ as well as their corresponding nanocomposites are composed of spinel oxides. According to crystal field theory,^95^ the transition metals in spinels can exhibit tetrahedral and octahedral coordinations, which result in different *d*-band splitting. The strong interaction between spinel bimetallic oxides with Ni-BDC MOF from one side and versatile valence states (Fe^2+^ → Fe^3+^ in Fe_3_O_4_ and Co^2+^ → Co^3+^ in ZnCo_2_O_4_) of spinel oxides from the other side allowed us to modify the electronic structure of resulting hybrids for further optimization of the binding conditions between the intermediates and reactants during the OER. However, NCF and its relevant nanocomposite stand out in terms of their OER performance. The above-written discussions showed the presence of layered Ni(OH)_2_ in these two samples (refer to the schematic image in Figure S1).

The layered structure-type oxides are categorized as the layered double hydroxides (LDH), and this family is usually cobalt- and nickel-based compounds on account of their remarkable OER activity. Subbaraman et al.^96^ reported a systematic investigation on the 3d TM (Ni, Co, Fe, and Mn) hydr(oxy)oxide for the OER and found the following trend for these compounds (Ni > Co > Fe > Mn). The excellent catalytic activity appertained to the optimal binding strength between Ni and OH. Besides, Corrigan^97^ demonstrated that a trace amount of Fe impurity would substantially increase the OER activity of NiOOH. They concluded that a further increase in the Fe content resulted in even higher performance. The overpotential, η^10^_OER_, observed in our work illustrated the same results. Among the ternary oxides, NCF [containing both iron oxide and Ni(OH)_2_] has the lowest value, followed by NCZ [containing only NiOOH] and NCC [neither iron oxide nor Ni(OH)_2_]. The OER performance for the nanocomposites is further elevated as coupling metal oxides/hydroxides with 2D MOFs endow the resulting hybrid with enormous active sites as a result of metal sites being highly exposed to electrolyte ions for use in catalytic reactions.

To study the electrochemical kinetic behavior, the Tafel slopes were calculated and plotted. As mentioned before, the Tafel slope is derived from polarization curves and measures how much potential is required to generate a 10-fold current density.^41^ To ensure the authenticity of the comparison, it is recommended to determine all Tafel slopes within a similar current density region. The results can be visualized in Figure 4b. Similar to OER experiments, Fe-containing materials possess more facile kinetics in terms of the Tafel slope. From Figure 4b, NCF/Ni-BDC and NCF displayed the lowest Tafel slopes of 35.1 and 39.5 mV dec^–1^. Based upon the previous reports, the proposed four-step OER process with relevant Tafel slopes is as follows:^98^
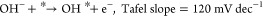
1
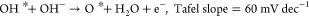
2
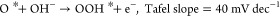
3
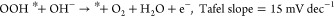
4

In the preceding reactions, * represents a catalytic site, and OH*, O*, and OOH* are the adsorbed intermediates.^73^ From the calculated values, the rate-determining step for NCF/Ni-BDC is between 40 and 15 mV dec^–1^, suggesting that the rate-determining step for this sample might be the formation of OOH* or desorption of O_2_ which is a sign of favorable kinetics. Similarly, the small Tafel slope (39.5 mV dec^–1^) of NCF implies that its OER kinetics conforms to OOH* formation. The rate-controlling step of NCZ/Ni-BDC and NCZ (Tafel slope = 60.2 and 68.7 mV dec^–1^, respectively), however, follows the second step in the OER process (O* formation).^99^Figure 4c illustrates the relation between the Tafel slope and overpotential at 10 mA cm^–2^ of the investigated electrocatalysts, demonstrating the superiority of NCF, NCZ, and their relevant nanocomposites.

For the Ni-based catalysts, the abovementioned reactions can be modified as^100−102^
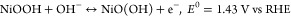
5
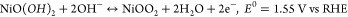
6
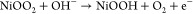
7
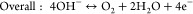
8

From these reactions, the first and second steps are reversible, whereas the third step is fast and irreversible, determining the overall rate of the process. Highly OER active catalysts are utilized to alleviate the kinetic barrier of the third step which is the case for NCF/Ni-BDC and NCF in this work. In general, NiO_6_ inside Ni-BDC can be oxidized to NiO_6_/NiOOH species as active sites to enhance the oxidation of OH^–^ to O_2_. Moreover, on the basis of XPS analysis, NCF/Ni-BDC nanocomposites contain metallic atoms with higher oxidation states and hence lower electron densities. Experimentally, Fe, Ni, and Co atoms within NCF/Ni-BDC draw more electrons compared to NCF and thereby are better electron acceptors.^64^ It must be borne in mind that Fe-based oxides/hydroxides can appreciably promote the OER performance once they are used together with Ni- and Co-based oxides/hydroxides which is the case for NCF/Ni-BDC and NCF electrodes. Concerning this, the XRD pattern of NCF (Figure S6) and HR-TEM micrographs of NCF/Ni-BDC (Figure 3b: third image from left) substantiated the coexistence of oxides/hydroxides. The identified crystalline interfaces for NCF/Ni-BDC nanocomposites are Ni(OH)_2_ (221) with Co_2_(OH)_2_CO_3_ (031) as well as spinel ferrite Fe_3_O_4_ (220) with Fe_2_O_3_ (110). According to previous studies^99^ on Co–Fe binary metal oxide, the interface formation between different oxides may minimize the activation barrier for the generation of intermediates. Concerning NCF and NCF/Ni-BDC electrodes, the existence of interfaces most likely alleviated the kinetic of OOH* formation to accelerate O_2_ generation.

In addition to the linear equation of Tafel discussed in the electrochemical measurements section, the Tafel slope can be inversely proportional to the charge-transfer coefficient (α) following the relation expressed below^95^
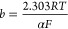
9

In this relation, *b*, *R*, *T*, and *F* are, respectively, indicative of the Tafel slope (V dec^–1^), the universal gas constant (8.314 J mol^–1^ K^–1^), absolute temperature (298 K), and Faraday constant (96485 A s mol^–1^). In this context, the higher the charge transfer ability of a catalyst, the smaller the Tafel slope. The computed charge transfer coefficient of each sample is tabulated in Table S3. In accordance with this table, NCF/Ni-BDC, NCF, and NCZ/Ni-BDC with respective α values of 1.68, 1.49, and 0.98 have the largest capacity for charge transfer, manifesting their good electrocatalytic kinetics. Comparing the charge-transfer ability of commercial RuO_2_ (0.54) with investigated materials in this study indicated that the as-prepared catalysts are much more competent at lower current densities and comparable to the RuO_2_ at higher current densities (see overpotential values at 50 mA cm^–2^ in Table S3).

To implement the overall water splitting with bifunctional catalysts, the HER is another equally significant half-reaction as the OER. Figure 4d portrays the HER performance of investigated samples together with commercial 20% Pt/C and blank NF. As expected, the state-of-the-art Pt/C@NF electrode and blank NF, respectively, featured the lowest and highest overpotential at 10 mA cm^–2^ (η^10^_HER_ = 61 and 314 mV). Among all the as-prepared materials, NCZ/Ni-BDC exhibited the lowest HER overpotential, η^10^_HER_ = 170 mV. In this sense, Ni-BDC, NCF, NCC, NCZ, NCF/Ni-BDC, and NCC/Ni-BDC, with respective η^10^_HER_ values of 268, 231, 183, 236, 215, and 215, showed inferior HER activity compared to NCZ/Ni-BDC. Interestingly, NCC stands out among the ternary mixed oxide catalysts owing to the incorporation of a Cu source. Cu, a TM element form d-block, displays high thermal and electrical conductivities, and its oxides and hydroxides have been widely studied for both OER and HER. What is more, all three Cu-based species [CuO, Cu(OH)_2,_ and Cu_2_(OH)_2_CO_3_] detected in the NCC sample (see Figure S6) have been known as excellent H_2_-production cocatalysts.^103−106^

To be consistent with the comparison basis of the OER performance, η^50^_HER_ was evaluated for each sample and is tabulated in Table S3. Comparison of the HER performance of fabricated electrodes at 50 mA cm^–2^ with that of commercial Pt/C@NF further elucidates the value of prepared catalysts. To this end, Pt/C@NF with η^50^_HER_ = 214 mV is still the best HER catalyst within the scope of this study, followed by NCZ/Ni-BDC@NF (351 mV), NCC@NF (366 mV), and NCF/Ni-BDC@NF (373 mV). At this point, it should be emphasized that coupling ternary mixed oxides with 2D Ni-BDC enhanced the HER performance of hybrids excluding the NCC/Ni-BDC electrode. The reason for this phenomenon can be pertained to lower active sites which can be explained by electroactive surface area. Finally, Table S2 presents the HER activity of NF-based MOFs and/or metal oxide electrocatalysts. With reference to this table, the best-performing nanocomposite in this work is comparable to other MOF-derived documented catalysts such as Fe–Ni@NC-CNT (η^10^_HER_ = 202 mV),^15^ Ni@NC-800 (η^10^_HER_ = 205 mV),^18^ and NiFe-MOF (η^10^_HER_ = 134 mV).^100^

In alkaline media, the HER kinetics is explained by the adsorption/desorption of hydrogen atoms/molecular hydrogen through either Volmer-Heyrovsky or Volmer-Tafel mechanisms, reaction^107^
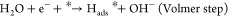
10
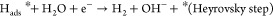
11
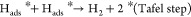
12where * stands for the available active sites and H_ads_* represents the atomic hydrogen at the active site. Ideally, one can infer the HER mechanism through Tafel plots derived from linear sweep voltammetry (LSV) curves. The experimental studies have already proven the corresponding Tafel slope values for each step. For this, if the reaction mechanism is controlled by the Volmer step, it requires a Tafel slope of ∼120 mV dec^–1^. In contrast, if the rate-controlling step of the HER is the Heyrovsky or Tafel reaction, a much smaller Tafel slope of about 40 mV dec^–1^ or 30 mV dec^–1^ will be uncovered. However, it should be stressed that the Tafel slope can be altered by several elements such as applied potential, the heavy presence of adsorbates, and mass transport in porous structures. Experimentally, it has been revealed that the Pt electrode is controlled by the Tafel path with a slope of 30 mV dec^–1^. Nevertheless, increasing applied potential leads to saturation of the catalyst surface with adsorbed hydrogen atoms, resulting in accelerated atom–atom recombination. Consequently, the rate-limiting step was flipped to a Volmer path with a Tafel slope of 120 mV dec^–1^.^107^ In this work, the Pt/C electrode showed a Tafel slope of 96.4 mV dec^–1^, verifying its validity with literature in alkaline conditions and approving its rate-limiting step, Volmer.

All the as-developed materials—except for NF—displayed a Tafel slope value beyond 120 mV dec^–1^ (see Figure 4e), indicating that they are controlled by the hydrogen atom adsorption step at the active sites. Still, among examined catalysts, NCZ/Ni-BDC exhibited a lower Tafel slope (137.2 mV dec^–1^) compared to other samples which confirms more facilitated kinetics of hydrogen adsorption at the active sites. Another important parameter which is an index to determine the intrinsic electrocatalytic activity of materials at the reversible overpotential (η = 0) is called exchange current density (*j*^0^),^2^ and it can be obtained by extrapolating the linear portion of the Tafel plot. The exchange current density is more often used as an activity parameter for the HER than that for the OER. The HER kinetics is facile regardless of the defined overpotential of the catalyst. In this sense, it can be stated that all reported catalysts have quite identical HER kinetics. This means that the exchange current density is directly correlated to the onset overpotential in the HER,^3^ the higher the *j*^0^, the more active the catalyst. Looking at the exchange current densities of the examined catalysts in Table S3 reveals that Pt/C (*j*^0^ = 1.08 mA cm^–2^) is the most active HER catalyst, and the NF substrate (*j*^0^ = 0.017 mA cm^–2^) is the least active HER catalyst in this study. In addition, in accordance with LSV results, NCZ/Ni-BDC (*j*^0^ = 0.78 mA cm^–2^) is the best HER catalyst among the as-synthesized materials, followed by NCC (*j*^0^ = 0.69 mA cm^–2^).

For decades, Ni-based alloys have been found as potential HER catalysts when combined with other TMs. For this, Raj et al.^108^ prepared several binary Ni-based alloys on mild-steel substrates for the HER using electrodeposition techniques. They found that NiZn alloy is one of the very best HER electrocatalysts. Moreover, Wang et al.^109^ reported that the addition of Zn to NiMo alloy could promote the valence states of metallic Ni and Mo and thereby significantly enhance the current density to 10- and 15-fold compared to pure Ni and NiMo alloy, individually. The best HER catalyst, NCZ/Ni-BDC, demonstrated significant enhancement compared to its components, Ni-BDC and NCZ. On the basis of XPS results, Ni, Co, and Zn atoms in the NCZ/Ni-BDC nanocomposite have lower oxidation states (= higher electron density) when compared to NCZ. This means that hydrogen atoms, H^+^, can easily adsorb at the active sites and facilitate the Volmer step. Another possibility is that NiO_6_ inside MOF can be partially reduced to form the Ni/NiO_6_ interface at the cathode. On such an interface, the OH^–^ generated by H_2_O splitting could preferentially attach to a NiO_6_ site at the interface due to strong electrostatic affinity to the locally positively charged Ni^2+^ species, while nearby Ni/Co/Zn sites would facilitate H^+^ adsorption and thus the Volmer process, imparting synergistic HER catalytic activity.^110^ Furthermore, as shown in Figure 3c (third image from left), the synergistic interface effect between crystalline phases in NCZ/Ni-BDC can be evinced. The partial reduction of metal oxide/hydroxides to metals can accelerate the proton adsorption and subsequent recombination to H_2_.^99^ Finally, Figure 4f represents the relation between Tafel slope and overpotential, suggesting that commercial Pt/C and NCZ/Ni-BDC are the best catalysts for the hydrogen production. In the meantime, we evaluated the OER and HER performances of NCZ’ [with Zn(NO_3_)_2_·6H_2_O as a zinc source] and NCZ’/Ni-BDC under the same conditions. Due to their poor performance (Figure S12) comparing to their counterparts, we decided not to conduct further experiments.

The electrochemical impedance spectroscopy (EIS) measurements were investigated at 0 V versus RHE to study the intrinsic charge-transfer capacity of the catalysts. Figure 4g illustrates the resulting Nyquist plots, curve-fitted with an equivalent circuit model to gain the charge-transfer resistance data. The equivalent circuit model for porous electrodes is represented as an inset of Figure 4g. In this model, *R*_e_, stands for electrolyte resistance, while *R*_p_/CPE1 and *R*_ct_ (charge-transfer resistance)/CPE2 are related to the porosity of the electrode and the interface between the catalyst and the electrolyte, respectively. The capacitive element can be replaced by a constant-phase element (CPE) due to the complex nature of the interfacial reaction.

Nyquist plots reveal a semicircle shape at high frequencies followed by a straight line at low frequencies, which in some plots tends to bend (NCZ/Ni-BDC and NCF/Ni-BDC). At high frequencies, the AC signal cannot penetrate the bottom of the pore because of the IR drop. On the other hand, at low frequencies, the AC signal can penetrate throughout the pore, and the measured capacitance corresponds to the total capacitance of the pore walls. What is more, at low frequencies, capacitance is the total capacitance of the porous and flat surface of the electrode. In practical applications, diffusion consists of a finite-length linear layer, where the diffusion layer corresponds to the thickness of the layer. Among the two types of finite-length diffusion (transmissive and reflective boundaries), transmissive boundaries are associated with steady-state concentration gradients in membranes, in which a semicircle is obtained because a DC can flow.^111^ Keeping these in mind, according to the Nyquist plots (Figure 4g), a transmissive boundary is shown because the arc region is included, and the sample is strictly capacitive, especially for the high-performance NCF/Ni-BDC and NCZ/Ni-BDC catalysts. On the contrary, for the NCZ, NCF*,* and Ni-BDC ones, the reflective boundary diffusion could be considered, in which the mass-transfer impedance displays a quite straight line at 45° at high frequencies because the penetration length of the AC signal is smaller than the layer thickness; consequently, the imaginary part goes to infinity as the constant current cannot flow in the system.

The intrinsic behavior of the catalysts on the kinetics of electrochemical processes could be interpreted from the data. The estimated values obtained from the EIS simulations by ZView software are listed in Table S4. Based on the results, the nanocomposite materials remarkably decrease surface resistance. In this sense, for the best-performing NCF/Ni-BDC and NCZ/Ni-BDC catalysts, respectively, the interface charge-transfer resistance (*R*_ct_) decreased to 116.3 and 126.9 Ω compared to the Ni-BDC (494.9 Ω), resulting in a significant improvement in electron transport. Undoubtedly, nanocomposite materials have more efficient electron transfer between the active sites of electrodes and electrolyte ions.

To further realize the remarkable performance of the two best materials toward OER (NCF/Ni-BDC) and HER (NCZ/Ni-BDC), the ECSAs of all samples were compared by estimating the double-layer capacitance (*C*_dl_) through cyclic voltammetry (CV) recorded at various scan rates within the potential window of 0.7–0.8 V versus RHE (Figure S13). In this context, ECSA can be obtained through the following equation^112^
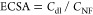
13where *C*_NF_ is a *C*_dl_ value of the NF substrate in 1.0 M KOH.^14^ Founded on the foregoing relation, ECSA and *C*_dl_ have direct relation, meaning that the higher the *C*_dl_, the higher the ECSA.^3^

Figure 4h illustrates the *C*_dl_ values for all samples that the following order can be drawn into attention: NCZ (0.121 mF cm^–2^; ECSA = 0.65) < NF (0.184 mF cm^–2^; ECSA = 1.0) < Ni-BDC (0.22 mF cm^–2^; ECSA = 1.2) < NCF (0.244 mF cm^–2^; ECSA = 1.32) < NCC/Ni-BDC (0.328 mF cm^–2^; ECSA = 1.78) < NCC (0.344 mF cm^–2^; ECSA = 1.87) < NCZ/Ni-BDC (0.483 mF cm^–2^; ECSA = 2.62) < NCF/Ni-BDC (0.525 mF cm^–2^; ECSA = 2.85). Interestingly, the electroactive surface areas for NCZ/Ni-BDC and NCF/Ni-BDC have enlarged over two-fold compared to bare Ni-BDC. Therefore, the best nanocomposite catalysts offer the largest exposed electroactive surface areas, confirming their enhanced electrocatalytic activity.^113^

Metal oxides/hydroxides are active catalytic centers for the OER, HER, and overall water splitting; however, they usually show compromised catalytic performance owing to limited exposed metal-active sites. In contrast, MOF material has inherent molecular metal centers as potential active sites for electrocatalysis.^100^ As mentioned in previous discussions, coupling metal oxides/hydroxides with 2D MOFs endows the resulting hybrid with enormous active sites as a result of metal sites being highly exposed to electrolyte ions for use in catalytic reactions. This was certified by a respective two-fold and four-fold increase of electrochemically active surface measured by using the double-layer capacitance of the composite electrodes, NCF/Ni-BDC (0.525 mF cm^–2^) and NCZ/Ni-BDC (0.483 mF cm^–2^) compared with their ternary oxide electrodes NCF (0.244 mF cm^–2^) and NCZ (0.121 mF cm^–2^). Moreover, as shown in Figure 1a, the layered structure of Ni-BDC allows for better storage and diffusion of electrolyte solution. However, it is accepted that ECSA alone is not an indicator to ascertain the activity of the electrode since ECSA is a quantitative factor to measure the active sites which means the quality of the active sites is equally important.^47^ To support the ECSA results, we determined the Brunauer–Emmett–Teller (BET) specific surface areas of Ni-BDC, NCF/Ni-BDC, and NCZ/Ni-BDC, which were found to be 3.27, 6.91, and 4.77 m^2^/g. Considering the low BET surface area of the samples which could be attributed to the closely packed lamellar morphology of the catalysts, their high performance can be stemmed from their in situ growth on the 3D microscopic porous framework of NF. The N_2_ adsorption–desorption isotherms can be found in Figure S14.

The stability and durability of the samples are other critical factors to appraise their performance for practical applications. The stability of the best-performing samples for the OER and HER was tested using the chronoamperometric experiments at 10, 20, and 50 mA cm^–2^. As shown in Figure 4j, the OER potential of NCF/Ni-BDC at 10, 20, and 50 mA cm^–2^ are fixed at 1.43, 1.47, and 1.59 V versus RHE, demonstrating outstanding long-term durability under 1.0 M KOH. Figure 4i depicts LSV curves toward OER for NCF/Ni-BDC before and after nearly 45 h continuous OER operation. Surprisingly, we observed a significant enhancement in the OER performance in terms of both overpotential at a fixed current and overall current density within the entire OER region. From the results before and after long-term durability experiments, η^10^_OER_ dropped from 120 to 80 mV and η^50^_OER_ declined from 450 to 200 mV. Besides, before the longevity test, the overall current density reached 80 mA cm^–2^ at 1.8 V versus RHE; however, the post-OER stability sample generated above 120 mA cm^–2^ at the same potential. Similarly, the HER long-term durability for NCZ/Ni-BDC was conducted in 1.0 M KOH. Unlike OER, NCZ/Ni-BDC exhibited decent HER durability. From Figure 4i, the LSV curve after 32 h HER operation uncovered that η^10^_HER_ and η^50^_HER_ increased from 170 to 190 mV and from 350 to 360 mV, respectively, verifying its relative durability. From Figure 4k, the chronoamperometry curve displayed the respective potential of 300, 385, and 520 mV at the fixed current density of 10, 20, and 50 mA cm^–2^.

#### 2.2.2 Post-Electrocatalysis Characterization

To investigate any type of alterations following the OER, HER, and their relevant prolonged water oxidation/reduction after roughly 45 and 32 h in alkaline conditions, we further characterized the catalysts. The microstructural studies for the high-performance OER (NCF and NCF/Ni-BDC) and HER (NCC and NCZ/Ni-BDC) samples were demonstrated via SEM-coupled EDS elemental mappings (Figures S15 and S16). Evidently, the samples after exposure to one HER did not display significant changes. Therefore, the morphology of HER samples remained largely intact and from the topographical image displayed in inset images of Figure S15b,d, no significant changes were observed. In contrast, the samples after exposure to one OER demonstrated some alterations. From the SEM image of NCF (Figure S15a), large crystalline features are noticeable which can be attributed to hundreds of nanorods joined to each other in an alkaline medium. The micrograph of NCF/Ni-BDC exhibited vertically aligned sheets like those before the OER reaction but thicker (see Figure S15c). Furthermore, the EDS elemental mappings confirmed the presence of all elements after electrocatalysis (Figure S16). Finally, XPS spectra were analyzed and assigned to distinctive species (refer to Figure S17).

**Figure 5:**
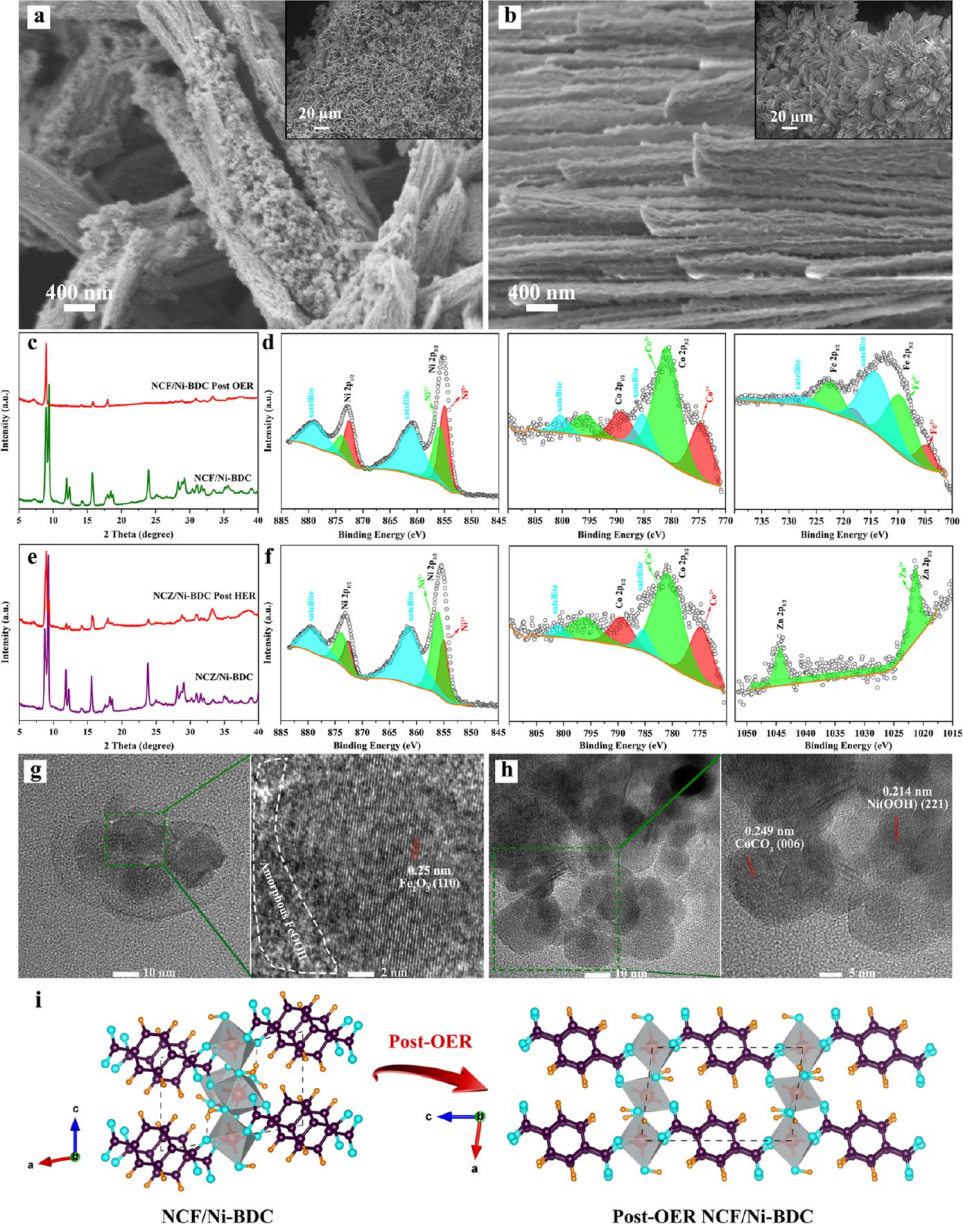
Post-electrolysis characterizations of NCF/Ni-BDC and NCZ/Ni-BDC after long-term durability tests. (a,b) SEM images of post-OER stability NCF/Ni-BDC and post-HER stability NCZ/Ni-BDC, (c,e) XRD patterns of NCF/Ni-BDC and NCZ/Ni-BDC before and after stability tests, respectively, (d) XPS Ni 2p, Co 2p, and Fe 2p spectra for NCF/Ni-BDC, (f) XPS Ni 2p, Co 2p, and Zn 2p spectra for NCZ/Ni-BDC, and (g,h) TEM and HR-TEM images of NCF/Ni-BDC and NCZ/Ni-BDC, respectively, and (i) crystal structure transformation after OER stability for the NCZ/Ni-BDC sample.

The high-resolution SEM image of the anodic catalyst after the long-term OER durability test can be seen in Figure 5a in which the lamellar structure changed to a great extent; heavily starched layers (Figure 1g) transformed to separate bundles of layers. The difference between the morphology of NCF/Ni-BDC before and after OER stability is significant in that the porosity within the structure increased tremendously (comparison between the inset image of Figure 5a and top-view SEM image of Figure S3b) and led to an extremely low overpotential at 50 mA cm^–2^ (200 mV). It can be observed that the porous space between each bundle is beyond several hundred nanometers. The major disadvantage of 2D MOF-derivative materials is regarded to be their potent inclination to aggregate which drastically declines their electrocatalytic activity.^64^ However, the inset image of Figure 5a shows that the aggregation of NCF/Ni-BDC has been significantly mitigated. This can be attributed to the crystal structure transformation in line with the reaction (14); from hydrated Ni_3_(OH)_2_(tp)_2_(H_2_O)_4_ within the triclinic system to anhydrous Ni_2_(OH)_2_tp (as shown in Figure 5i) within the monoclinic system (space group *C*2/*m*) which is supported by the XRD pattern of NCF/Ni-BDC after the OER durability test (see Figure 5c).^52^ In addition, from the TEM images, the original shape of nanoparticles cannot be found. Figure 5g shows the HR-TEM images of the post-OER anode, in which the formation of amorphous phases (delineated by a white dashed line) such as FeOOH is evident which is due likely to the partial degradation of Fe_2_O_3_ with a lattice fringe of 0.25 nm corresponding to the crystalline plane of (110). Eventually, the HR-TEM image, as shown in Figure S18a, disclosed the existence of NiOOH. It must be noted that NiOOH as the actual catalytic active site for the OER can be created during electrochemical activation following the oxidation of Ni(OH)_2_.^47,114^
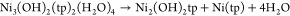
14

On the flip side, the cathodic catalyst remained largely unchanged both morphologically and structurally. Figure 5e depicts the XRD pattern of the post-HER catalyst, reflecting all diffraction peaks matching with the XRD pattern of the fresh sample. Moreover, the SEM photograph is shown in Figure 5band illustrated no microstructural and morphological changes after 32 h of continuous HER experiment. Furthermore, the TEM and HR-TEM analyses (Figure 5h) confirmed the presence of embedded nanoparticles with high crystallinity, identical to the fresh catalyst, NCZ/Ni-BDC (see Figure S18b). With this in mind, it can be inferred that partial detachment of the catalyst from its substrate resulted in a slightly higher overpotential after 32 h. The compositional survey and chemical states for the post-OER and HER catalysts were implemented. Concerning the NCF/Ni-BDC after long-term OER (see Figure 5d), the high-resolution XPS core-level spectra for Ni 2p revealed the two doublets corresponding to Ni^2+^ and Ni^3+^ and meaningfully shifted to small BEs compared to the fresh sample (refer to Figure 2d), implying that low-priced nickel would be oxidized to high-priced nickel in the reaction process.^56^ The same situation occurred for Co 2p and Fe 2p for which both 2+ and 3+ states appeared and shifted to small BEs. In addition, the comparison of Fe^2+^ before and after prolonged OER discloses a drastic intensity decrease, which can be the coverage effect of the amorphous phases such as the FeOOH layer on the surface.^115^ Finally, the XPS analysis of Ni 2p, Co 2p, and Zn 2p (as shown in Figure 5f) slightly shifted in the direction of small BEs as opposed to the fresh sample (Figure 2f), manifesting the formation of new species and in turn change in the electronic configuration of metal atoms on the surface. It can be concluded that the cathodic side did not go under severe change, and no metal Ni, Co, and Zn were formed during the reduction process.

We need to clarify the complicated nature of the post-OER XPS results. A thorough investigation of the OER dynamics of Ni–Fe mixed by Görlin et al.^116^ suggested that Ni-based catalysts undergo a noticeable redox state between 2+ and 3+/4+ following OER electrolysis, corroborated by both X-ray absorption spectroscopy (XAS) and operando differential electrochemical mass spectrometry (DEMS)-based faradaic efficiency data. However, they stated that the story differs when Fe comes into the picture. The presence of Fe in the active mixed Ni–Fe catalysts stabilizes the low-valent Ni centers. Moreover, pure Fe likewise did not display a change in its redox state. From the redox state point of view, concerning XPS results for NCF/Ni-BDC, our work is in line with the study conducted by Görlin et al.^116^

To further prove the stability of the catalysts after OER and HER longevity tests, the amount of dissolved metallic atoms was recorded via inductively coupled plasma–mass spectrometry (ICP–MS). As shown in Table S5, only a trace amount of elements are present in the electrolyte, meaning that the most of elements are preserved after long-term OER/HER durability.

#### 2.2.3 Overall Water Splitting

**Figure 6:**
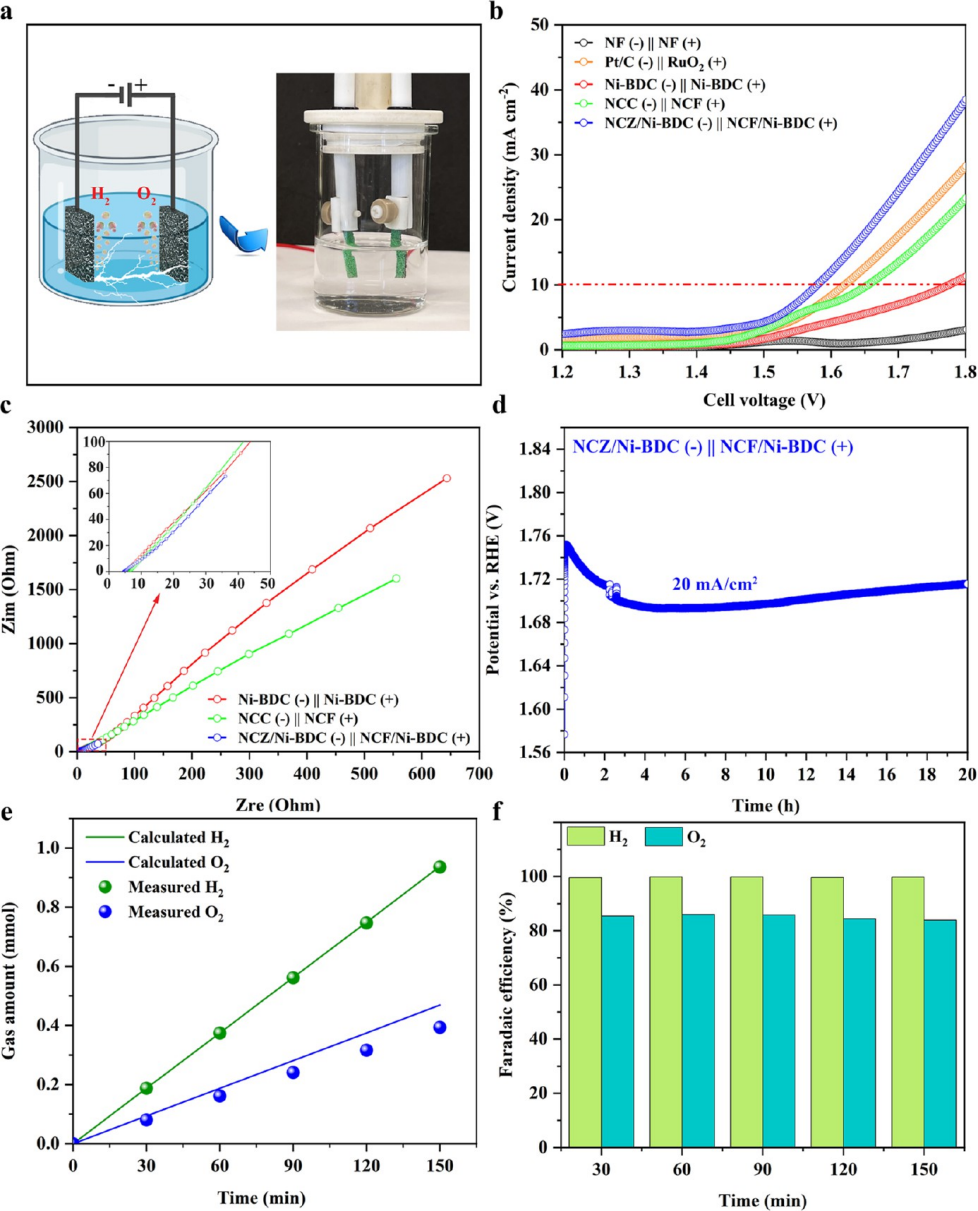
Electrocatalytic activity of various electrolyzers for overall water splitting in 1.0 M KOH. (a) Schematic and a digital photograph of a two-electrode cell used in this work, (b) polarization curves of various catalysts used as the cathode (−) and anode (+) in a two-electrode cell, (c) EIS spectra recorded at the cell potential of 0 V for constructed electrolyzers, (d) long-term durability test of the best-performing NCZ/Ni-BDC ∥ NCF/Ni-BDC couple at the constant current density of 20 mA cm^–2^, (e) electrocatalytic efficiency of overall water splitting for H_2_ and O_2_ production at *j* = 20 mA cm^–2^ on NCZ/Ni-BDC ∥ NCF/Ni-BDC electrodes, and (f) quantification of the Faraday efficiency of water electrolysis for the NCZ/Ni-BDC ∥ NCF/Ni-BDC electrolyzer.

Given the remarkable OER performance of NCF/Ni-BDC and the great HER performance of NCZ/Ni-BDC in 1.0 M KOH solution, we further assembled a couple-electrode cell to examine its competence in overall water splitting. For comparison, NF ∥ NF, Pt/C (cathode) ∥ RuO_2_ (anode), Ni-BDC ∥ Ni-BDC, and NCC (cathode) ∥ NCF (anode) electrolyzers were likewise constructed and tested under the same conditions (see Figure 6a). From Figure 6b, NCZ/Ni-BDC ∥ NCF/Ni-BDC delivered 10 mA cm^–2^ at the applied cell voltage of 1.58 V which is 20 mV lower than that of benchmark Pt/C ∥ RuO_2_. Even though Pt/C is the best HER material in this work (compared to the as-prepared catalysts), the presence of NCF/Ni-BDC as the best-performing OER sample (much better than RuO_2_) controls the two-electrode cell performance. Besides, the performance of the assembled couple is comparable to the recently documented couple electrode cells (Table S2). In addition, NF ∥ NF exhibited an insignificant performance and was not able to generate 10 mA cm^–2^ within the applied potential window (1.2–1.8 V), demonstrating the negligible impact of the NF substrate on the electrocatalytic performance of the samples. Moreover, Ni-BDC ∥ Ni-BDC and NCC ∥ NCF couples afforded 10 mA cm^–2^ at the respective cell voltage of 1.77 and 1.65 V. The EIS experiments were carried out on the assembled electrolyzers at the cell potential of 0 V (fitted with the same equivalent circuit model of Figure 4g), and the results confirmed the significant difference between NCZ/Ni-BDC ∥ NCF/Ni-BDC with other electrolyzers, showing appreciably lower resistance (refer to Figure 6c). The related fitted values are listed in Table S4. Based on the results, for a couple-electrode cell, the Nyquist plots displayed more reflective boundary diffusion almost at all frequencies. For the Ni-BDC ∥ Ni-BDC and NCC ∥ NCF couples, the plots did not include the complete arc region, and the samples were not utterly capacitive. On the other hand, for the NCZ/Ni-BDC ∥ NCF/Ni-BDC system, the interface charge-transfer resistance (*R*_ct_) decreased to 423 Ω compared to the Ni-BDC ∥ Ni-BDC (37.8 × 10^9^ Ω) and NCC ∥ NCF (2818 Ω), resulting in a significant improvement in electron transport for the nanocomposite system.

To analyze the practicality of the NCZ/Ni-BDC ∥ NCF/Ni-BDC in terms of durability and stability, the current density of 20 mA cm^–2^ was applied to the couple-electrode cell for 20 h. The chronopotentiometry curves in Figure 6d disclose that NCZ/Ni-BDC ∥ NCF/Ni-BDC couple is quite stable after 20 h in 1.0 M KOH medium, exhibiting a negligible increase in the required potential to reach 20 mA cm^–2^ throughout the whole 20 h. Furthermore, the amount of H_2_ and O_2_ from the overall water splitting (without using any membrane to separate the gas products) operated at 20 mA cm^–2^ for 150 min was quantified by a gas chromatograph (GC), as shown in Figure 6e. The faradaic efficiencies for both HER and OER reactions were calculated in accordance with eq 15 and compared to the theoretical data^117^ (refer to Figure 6f).
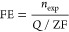
15

In the preceding formula, *n*_exp_ refers to the number of moles of H_2_ or O_2_ experimentally produced at constant current density (e.g., 20 mA cm^–2^), *Q* (C) denotes the total amount of charge, *Z* is the number of transferred electrons (i.e., in HER, *Z* = 2 and in OER, *Z* = 4), and *F* is the Faraday’s constant (96,485 C mol^–1^). It was found that HER and OER have, respectively, the efficiencies of 100 and 85%, demonstrating great potential for electrolytic hydrogen production.

To learn the plausible reasons for the 15% loss in O_2_ faradaic efficiency, we should take the reaction dynamics of the OER process into account which was described by Görlin et al.^116^ According to their operando DEMS analysis which was applied to quantitatively calculate the O_2_ faradaic efficiency during a CV OER scan, the total efficiency is a result of two components: (i) the faradic charge which accounts for the number of oxidizing species depleted for water oxidation (O_2_ formation) and (ii) the oxidation charge which accounts for the number of redox species needed for oxidation-state changes of the metal centers. Based on this study which was conducted on Ni-based, Ni–Fe mixed, and Fe-based catalysts, the low faradaic O_2_ evolution for Ni-based materials was ascribed to the one-electron Ni^2+/3+^ redox charge process. They further stated that the addition of Fe into Ni catalysts could stabilize the redox properties of Ni centers, leading to higher O_2_ faradaic efficiencies in Ni–Fe mixed materials. From this, we can conclude that a 15% loss in OER faradaic efficiency for NCF/Ni-BDC can be originated from the redox process during water electrolysis of metal centers, especially Ni which is the most dominant metal in this framework relative to Fe and Co. Still, it is not possible to claim the precise oxidation states of Ni, Fe, and Co in this material after the electrolysis process which contributed to the faradaic efficiency loss.

#### 2.2.4 Mechanism Discussion

First, the hierarchical growth of NCM/Ni-BDC@NF brought about the synergy between the three components. Multiple metal centers created synergy in NCM oxides, and their peculiar morphology strengthened the synergistic effect between 2D layers of Ni-BDC and NCM. Besides, the 3D open structure of NF with a large specific surface area improved the mass and electron transfer of the in situ grown materials. Second, the crystal/crystal interfaces formed within the 3D structure of NCM promoted the adsorption/desorption of the intermediates. Third, on the anode side, NiO_6_ inside Ni-BDC can be oxidized to NiO_6_/NiOOH species as active sites to enhance the oxidation of OH^–^ to O_2_. Moreover, the strong interactions between Ni-BDC and NCF could decrease the electron density and generate Ni, Co, and Fe with higher oxidation states which greatly boosted the intrinsic OER activity of NCF/Ni-BDC. Fourth, on the cathode side, NiO_6_ inside MOF can be partially reduced to form the Ni/NiO_6_ interface. On such an interface, the OH^–^ generated by H_2_O splitting could preferentially attach to a NiO_6_ site at the interface due to strong electrostatic affinity to the locally positively charged Ni^2+^ species, while nearby Ni/Co/Zn sites would facilitate H^+^ adsorption. Furthermore, the interaction between Ni-BDC and NCZ generated Ni, Co, and Zn with higher electron densities and encouraged the electrolytic HER of NCZ/Ni-BDC. In addition to the above-written aspects, the layered structure of Ni-BDC facilitated the storage and diffusion of electrolyte solution to deposited NCM nanoparticles. Last, but not least, coupling metal oxides/hydroxides with 2D MOFs endows the resulting hybrid with enormous active sites as a result of metal sites being highly exposed to electrolyte ions for use in catalytic reactions. This was certified by an increase in electrochemical active surface measured by using the double-layer capacitance of the composite electrodes. Altogether, all the mentioned features of fabricated hybrids are responsible for remarkable OER, HER, and overall water splitting.

## 3 Conclusions

In conclusion, we successfully fabricated Ni–Co-M oxide (M = Fe, Cu, and Zn) [NCM:M = F, C, and Z]/Ni-BDC MOF@NF nanocomposites on the conductive 3D porous backbone of NF via a simple solvothermal process. Compared to Ni-BDC and ternary oxide, NCF/Ni-BDC and NCZ/Ni-BDC were endowed with higher electrochemically active surface area. The oxidation states of the metallic atoms in both nanocomposites were manipulated in favor of OER and HER. In this context, NCF/Ni-BDC demanded respective potential of 1.35 and 1.68 V versus RHE to deliver 10 and 50 mA cm^–2^ toward OER. At the same time, NCZ/Ni-BDC yielded 10 and 50 mA cm^–2^ at the respective overpotential of 170 and 350 mV toward HER in alkaline solutions. When used for overall water splitting, the NCZ/Ni-BDC ∥ NCF/Ni-BDC couple electrode only required a cell voltage of 1.58 V to achieve the current density of 10 mA cm^–2^, outperforming the benchmark electrode couple of Pt/C ∥ RuO_2_. The NCF/Ni-BDC nanocomposite was decorated with multiple crystalline interfaces such as Ni(OH)_2_ (221) with Co_2_(OH)_2_CO_3_ (031) as well as spinel ferrite Fe_3_O_4_ (220) with Fe_2_O_3_ (110), which endowed the anode with the remarkable performance for the adsorption of OH^–^ ions to facilitate the oxygen-evolving process. On the cathodic side, the Ni-BDC nanosheets were decorated with ZnCo_2_O_4_ (222) and CoCO_3_ (006), which in turn led to lower oxidation states that expedited the H^+^ ion adsorption. Finally, the as-prepared couple electrolyzer demonstrated excellent durability at an applied cell potential of 20 mA cm^–2^ for 20 h. As a whole, the synergy between multiple factors including the hierarchical architecture of Ni-BDC nanosheets and ternary oxide nanoparticles on the 3D open framework of the conductive substrate, modulation of electronic structure, formation of multiple metal/metal oxide interfaces, and creation of abundant active sites culminated in the design of highly efficient catalysts toward overall water splitting. The faradaic efficiencies of the NCZ/Ni-BDC ∥ NCF/Ni-BDC electrolyzer were obtained by quantifying the H_2_ and O_2_ via a gas chromatograph and were estimated to be 100 and 85%, respectively.

## 4 Experimental Section

### 4.1 Catalyst Fabrication

#### 4.1.1 Materials

1,4-benzenedicarboxylic acid [terephthalic acid/1,4-BDC, Sigma-Aldrich, 98%], *N*,*N*-dimethylformamide [DMF, Sigma-Aldrich], nickel nitrate hexahydrate [Ni(NO_3_)_2_·6H_2_O, Sigma-Aldrich, 99.999% trace metal basis], cobalt nitrate hexahydrate [Co(NO_3_)_2_.6H_2_O, Sigma-Aldrich, 99.999% trace metal basis], iron nitrate nonahydrate [Fe(NO_3_)_3_·9H_2_O, Sigma-Aldrich, ≥99.95% trace metals basis], zinc hydroxide nitrate [Zn(OH) (NO_3_)·H_2_O], zinc nitrate hexahydrate [Zn(NO_3_)_2_.6H_2_O, Sigma-Aldrich, 98%], copper nitrate trihydrate [Cu(NO_3_)_2_·3H_2_O, Sigma-Aldrich, 99.999% trace metals basis], and urea [CH_4_N_2_O, Sigma-Aldrich, ≥98%] were utilized without any prior treatment.

#### 4.1.2 Synthesis of Ni-BDC@NF

The commercial NF with a size of 2 cm × 3 cm was ultrasonically treated with 2.0 M HCl solution, acetone, deionized (DI) water, and ethanol each for 10 min to get rid of impurities such as oxide on the NF surface and dried in the open air. To grow Ni-BDC on NF, two solutions were simultaneously prepared by dissolving 5 mmol 1,4-BDC in 15 mL of DMF (solution 1) and 5 mmol Ni(NO_3_)_3_·6H_2_O in 15 mL of DI water (solution 2) under vigorous stirring for 30 min. Thereafter, solution 1 was mixed with solution 2, and they were stirred for another 30 min. The homogeneous resulting solution was then ultrasonicated for 60 min and transferred to a 50 mL Teflon-lined stainless-steel autoclave containing a pretreated piece of NF. The sealed batch was kept at 180 °C for 24 h and cooled naturally to room temperature. The obtained product was washed thoroughly with DMF, DI water, and ethanol and vacuum dried overnight at 70 °C.

#### 4.1.3 Synthesis of Ni–Co-M Oxide (M = Fe, Cu, and Zn)@NF

For the growth of Ni–Co-M oxide (M = Fe, Cu, and Zn) on NF, a simple one-step hydrothermal procedure was adopted. For this, a specified amount of Ni(NO_3_)_2_.6H_2_O (1 mmol), Co(NO_3_)_2_.6H_2_O (1 mmol), metal nitrate hydrate [Fe(NO_3_)_3_·9H_2_O (1 mmol) or Cu(NO_3_)_2_·3H_2_O (1 mmol) or Zn(OH) (NO_3_)·H_2_O (1 mmol)], and CH_4_N_2_O (8 mmol) were added to a 100 mL beaker containing 40 mL of DI water and stirred for 2 h to receive precursor solution. The solution was then transferred to a 50 mL Teflon-lined hydrothermal autoclave in which a piece of NF was placed. The autoclave system was sealed and maintained at 180 °C for 10 h and quenched naturally to room temperature. The resultant sample was rinsed several times with DI water, and ethanol and was dried overnight in a vacuum oven at 60 °C. The mass loading of Ni–Co–M oxide@NF was <5 mg cm^–2^. At this point for convenience, the labeling of the nanocomposites is regarded as NCF, NCC, and NCZ for respective nanocomposites of Ni–Co–Fe oxide, Ni–Co–Cu oxide, and Ni–Co–Zn oxide. It is worth noting that we prepared another sample as NCZ′ with Zn(NO_3_)_2_.6H_2_O as a zinc source through the above-written procedure and performed the electrochemical measurements. Due to the poor electrocatalytic activity of NCZ′, we did not conduct further experiments.

#### 4.1.4 Synthesis of Ni–Co–M (M = Fe, Cu, and Zn) Oxide/Ni-BDC@NF Nanocomposites

For the fabrication of Ni–Co–M oxide/Ni-BDC on NF, the same procedure as for the growth of Ni-BDC@NF was followed. Once the stirring of solution 1 + solution 2 was completed, a certain amount of already prepared powder of Ni–Co–M (15 wt %) was added to the solution mixture and stirred for 60 min. The solution was further mixed by ultrasonic vibrations for another 60 min. The precursor solution was transferred to a 50 mL Teflon-lined autoclave reactor and heated at 180 °C for 24 h. Following the completion of the reaction, the treatment of the produced materials was identical to that of Ni-BDC@NF. The mass-loading of Ni–Co–M oxide/Ni-BDC@NF was about 10 mg cm^–2^. To preclude redundancy, from this point, the following labeling is considered; NCF/Ni-BDC@NF, NCC/Ni-BDC@NF, and NCZ/Ni-BDC@NF for respective nanocomposites of Ni–Co–Fe oxide/Ni-BDC@NF, Ni–Co–Cu oxide/Ni-BDC@NF, and Ni–Co–Zn oxide/Ni-BDC@NF.

Figure S1 schematically presents the coupling process of Ni(OH)_2_ as one of the dominant phases in NCF with the Ni-BDC ligand. As the DMF solvent is slightly acidic, Ni(OH)_2_ partially loses its hydroxyl groups under sonication, resulting in Ni^δ+^ and OH^δ−^. Then, OH^δ−^ attracts the protons (H^+^) of the BDC ligand to release the H_2_O molecule. Next, deprotonated BDC ligands can react with unsaturated Ni^δ+^ to form Ni(OH)_2_/Ni-BDC. Similar interactions can take place for other phases to create heterostructures between Ni-BDC and ternary mixed oxides.

#### 4.1.5 Preparation of the RuO_2_@NF and Pt/C@NF Electrocatalysts

Commercial RuO_2_ (5 mg) was dispersed in a 1000 μL solution of ethanol and DI water (700:300) and sonicated for 30 min followed by the addition of 20 μL of Nafion solution with another 30 min sonication to achieve a homogeneous ink. Similarly, 20% Pt/C (5 mg) was sonicated for 30 min in a 1000 μL solution of ethanol and DI water (600:400), and 20 μL of Nafion solution was added to the solution with 30 min further sonication. The prepared ink was dipped on a piece of NF to receive a mass loading of about ∼5 mg cm^–2^.

### 4.2 Catalyst Characterization

The crystalline planes of the resultant powders were identified by a Rigaku Mini Flex 600 X-ray diffractometer, Cu Kα radiation (λ = 1.5418 Å). XRD patterns were recorded in the 2θ range of 5–70° at a scanning rate of 5° s^–1^. The morphology of the catalysts grown on the NF was characterized by a field emission SEM (FESEM; Zeiss Ultra Plus) connected to a Bruker Xflash 5010 (123 eV spectral resolution) EDS detector. The quantitative concentrations of TMs within the structure of ternary mixed oxides were characterized by a BRUKER TIGER S8 wavelength-dispersive X-ray fluorescence spectrometer. The microstructure of the samples scraped off the NF was in addition examined by TEM; Thermo Scientific Talos F200S 200 kV TEM). The SAED image was obtained via a Thermo Scientific Talos F200S 200 kV TEM instrument. Besides, the lattice fringes of the revealed nanoparticles were specified by the HR-TEM photographs taken by the same apparatus. In addition, HAADF-STEM images and the associated EDS elemental mappings were conducted on a Hitachi HT7800 (TEM) with EXALENS (120 kV) working at the HR mode. To determine the thickness of a single nanosheet, AFM was carried out using Bruker Dimension Icon AFM. The surface compositions of the powdered samples were analyzed on a Thermo Scientific K-Alpha through XPS with an Al Kα monochromator source (1486.6 eV). The deconvolution of the XPS spectra was carried out through Avantage 5.9 software, and all spectra were corrected according to the BE of C 1s = 284.5 eV. To distinguish the functional groups, a JASCO 6800 FTIR full vacuum & FTIR microscope apparatus was employed, and the spectra were recorded in the wavenumber region of 4000 to 400 cm^–1^. To determine the BET specific surface area of the powder products through N_2_ adsorption–desorption isotherms, a Micromeritics ASAP 2010 instrument was exploited. Finally, ICP–MS Agilent 7700x was conducted to study the trace amount of dissolved TMs in an alkaline solution for post-stability tests.

### 4.3 Electrochemical Characterization

The electrochemical studies were performed on an AutoLab Potentiostat Galvanostat in a typical three-electrode system using catalysts grown on NF (0.5 cm × 1 cm) as working electrodes, Pt spring as the counter electrode, and RHE; HydroFlex as the reference electrode in alkaline conditions (1.0 M KOH). The LSV curves were recorded at a scan rate of 5 mV s^–1^ to evaluate the electrocatalytic performance. The OER overpotentials at a fixed current density (*j*) were calculated based on
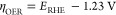
16within the OER potential window (1.2–1.8 V), and the HER overpotentials were demonstrated according to
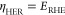
17in the HER potential region (0 to −1 V). The Tafel slopes were achieved by replotting the LSV curves, η as a function of log(*j*) via the Tafel equation
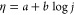
18where *b* is the Tafel slope^2^

The OER and HER longevity experiments were carried out via chronopotentiometry at a fixed *j* of 10, 20, and 50 mA cm^–2^. EIS was recorded at the potential of 0 V with a sinusoidal amplitude of 10 mV at a frequency ranging from 100 kHz to 0.1 Hz. The ECSA was acquired through the *C*_dl_ with respect to the CV measured at various scan rates of 80, 100, 120, 140, 160, 180, and 200 mV s^–1^ across the potential window from 0.7 to 0.8 V versus RHE. All the electrochemical data were presented without iR correction. Finally, gas products (H_2_/O_2_) for the full-cell water electrolysis were detected and quantified using a gas chromatograph (7820A, GC-System, Agilent) equipped with a thermal conductivity detector.

## Supporting Information Available

Comprehensive chemical and electrochemical characterization data including all the extra SEM/EDS images before and after electrochemical reactions, XRF numerical results, TEM/HR-TEM micrographs, XRD patterns, FT-IR spectra, ECSA, electrochemical performance, and BET adsorption–desorption isotherms (PDF)
Overall water splitting with best-performance catalysts in this work (MP4)
