# Supplementary material for: Designing In Situ Grown Ternary Oxide/2D Ni-BDC MOF Nanocomposites on Nickel Foam as Efficient Electrocatalysts for Electrochemical Water Splitting
Source: ACS Mater Au. 2022 Dec 28;3(2):143–63. doi: 10.1021/acsmaterialsau.2c00073 (PMC9999482; doi:10.1021/acsmaterialsau.2c00073)
Supplement: Supplementary file 1 — mg2c00073_si_001.pdf [file mg2c00073_si_001.pdf]

Supporting Information

Designing In Situ Grown Ternary Oxide/2D Ni-BDC MOF Nanocomposites on Nickel Foam as Efficient Electrocatalysts for Electrochemical Water Splitting

Ebrahim Sadeghi[1,2], *Naeimeh Sadat Peighambardoust*[1], *Sanaz Chamani*[1], *and Umut Aydemir*[1,3,]*

[1] Koç University Boron and Advanced Materials Applications and Research Center (KUBAM), Sariyer, Istanbul, 34450, Turkey

[2] Graduate School of Sciences and Engineering, Koç University, Sariyer, Istanbul, 34450, Turkey

[3] Department of Chemistry, Koç University, Sariyer, Istanbul, 34450, Turkey

* Corresponding author; Email: uaydemir@ku.edu.tr

Video S1. Overall water splitting with a high gas-evolution rate.

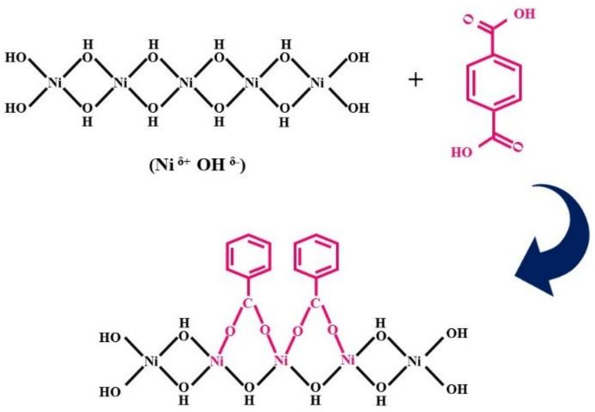

Figure S1. The coupling mechanism of $Ni(OH)_2$ as the dominant phase of NCF with Ni-BDC ligand.

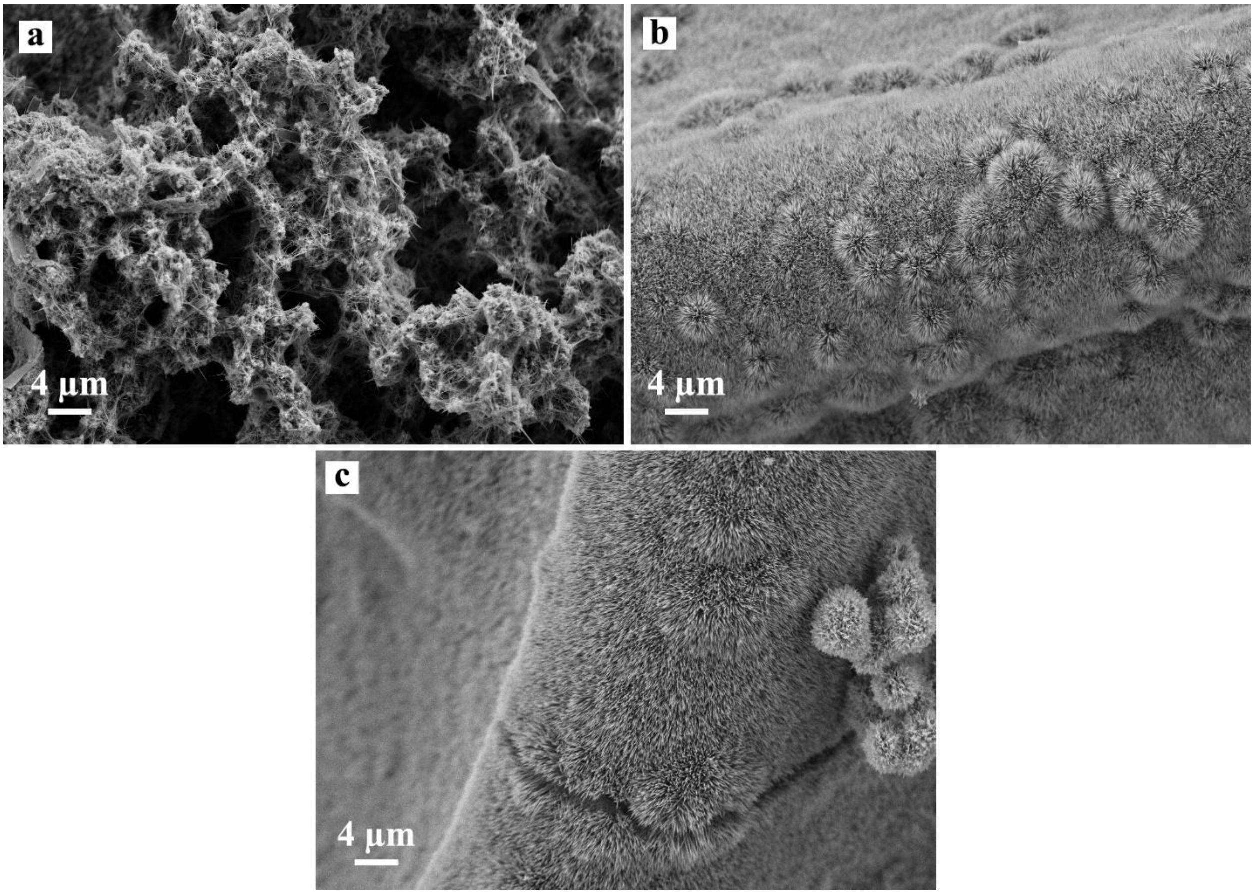

Figure S2. SEM micrographs of a) NCF@NF, b) NCC@NF, and c) NCZ@NF grown on NF backbone with low magnifications.

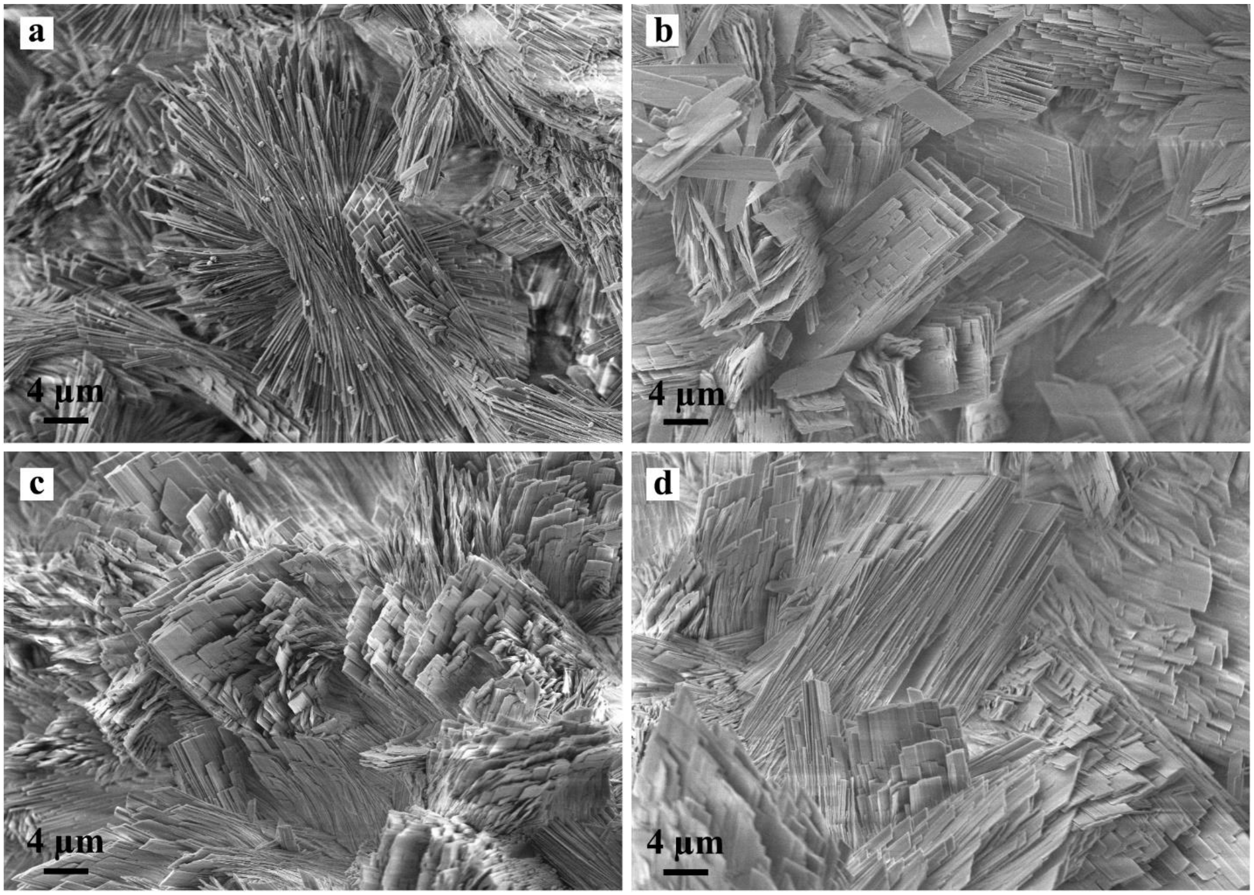

Figure S3. Top-view SEM images of a) Ni-BDC@NF, b) NCF/Ni-BDC@NF, c) NCC/Ni-BDC@NF, and d) NCZ/Ni-BDC@NF.

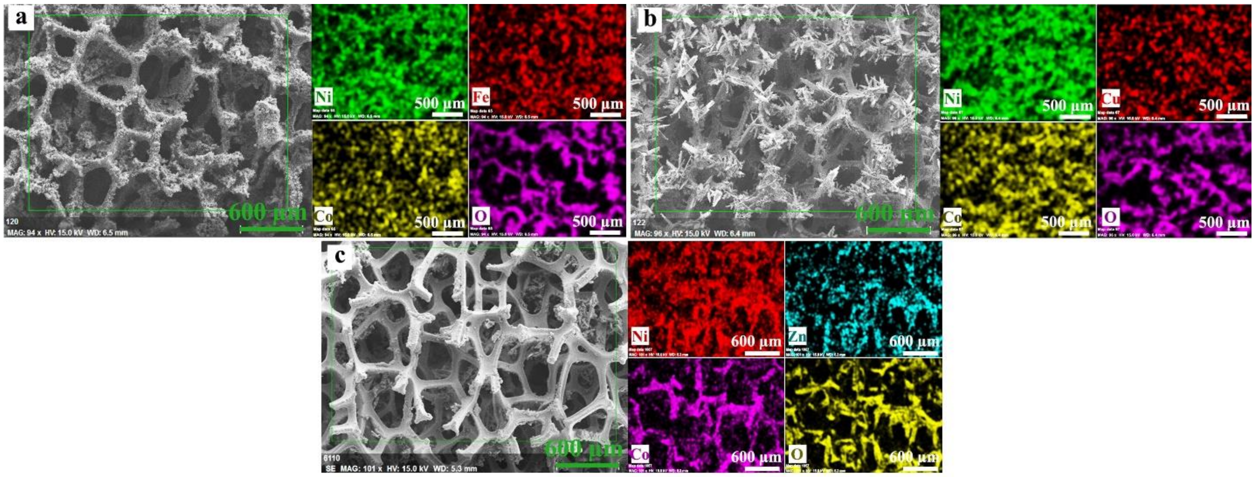

Figure S4. SEM/EDS elemental mappings of a) NCF@NF, b) NCC@NF, and c) NCZ@NF; a very large area selected to observe the distribution of elements throughout the NF.

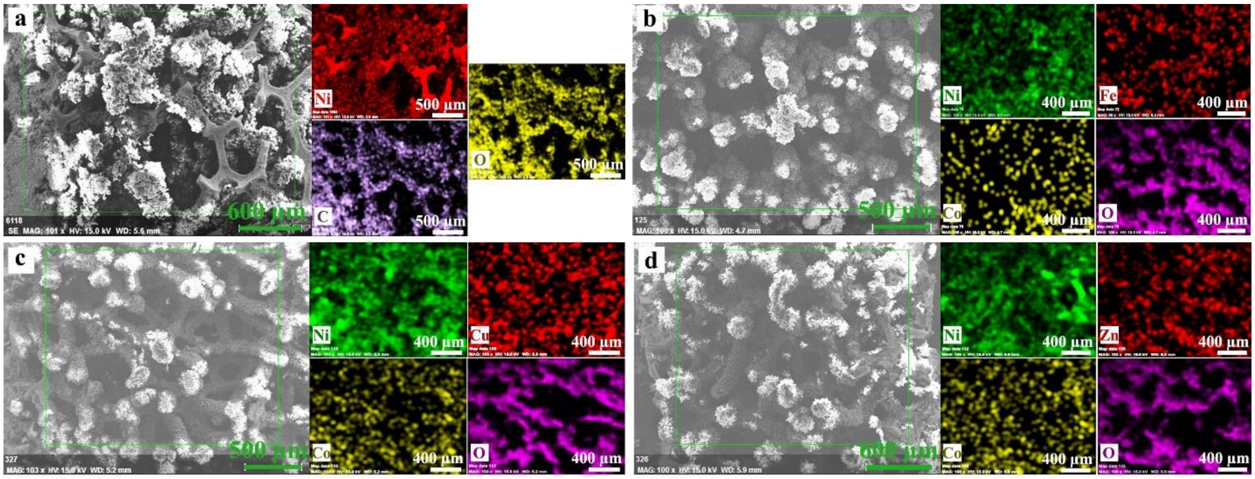

Figure S5. SEM/EDS elemental mappings of a) Ni-BDC@NF, b) NCF/Ni-BDC@NF, c) NCC/Ni-BDC@NF, and d) NCZ/Ni-BDC@NF; a very large area selected to observe the distribution of elements throughout the NF.

Table S1. The quantitative results of XRF analysis of ternary mixed oxides.

Sample	Elements	Line 1	Concentration (wt%)	Line 2	Concentration (wt%)
NCF	Ni	Ni Kα1	43.34	Ni Kβ1	42.18
	Co	Co Kα1	28.33	Co Kβ1	27.18
	Fe	Fe Kα1	28.33	Fe Kβ1	30.64
NCC	Ni	Ni Kα1	46.55	Ni Kβ1	42.34
	Co	Co Kα1	34.65	Co Kβ1	38.46
	Cu	Cu Kα1	18.80	Cu Kβ1	19.20
NCZ	Ni	Ni Kα1	40.70	Ni Kβ1	38.86
	Co	Co Kα1	24.30	Co Kβ1	25.83
	Zn	Zn Kα1	35.00	Zn Kβ1	35.31

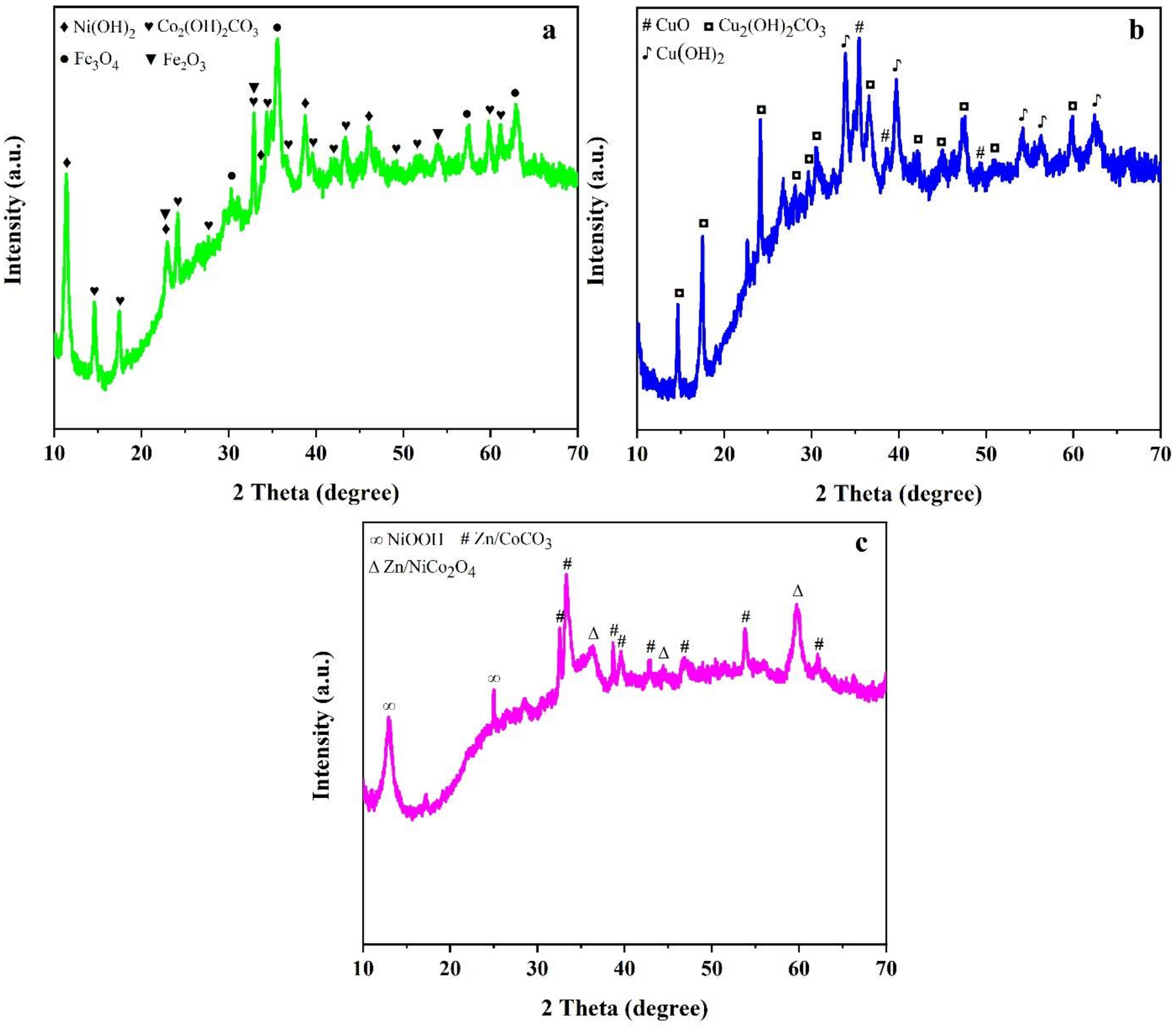

Figure S6. XRD patterns of various ternary oxides, NCM (M = F, C, and Z) stripped off from NF.

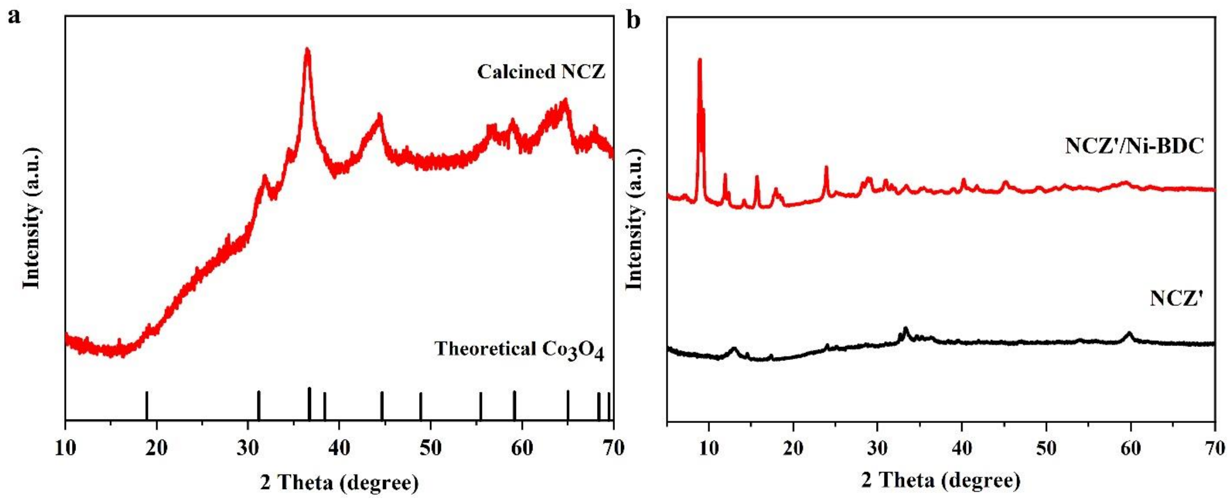

Figure S7. XRD patterns of a) Calcined NCZ catalyst sample and b) Ternary oxide NCZ’ and its corresponding nanocomposite NCZ’/Ni-BDC stripped off from NF.

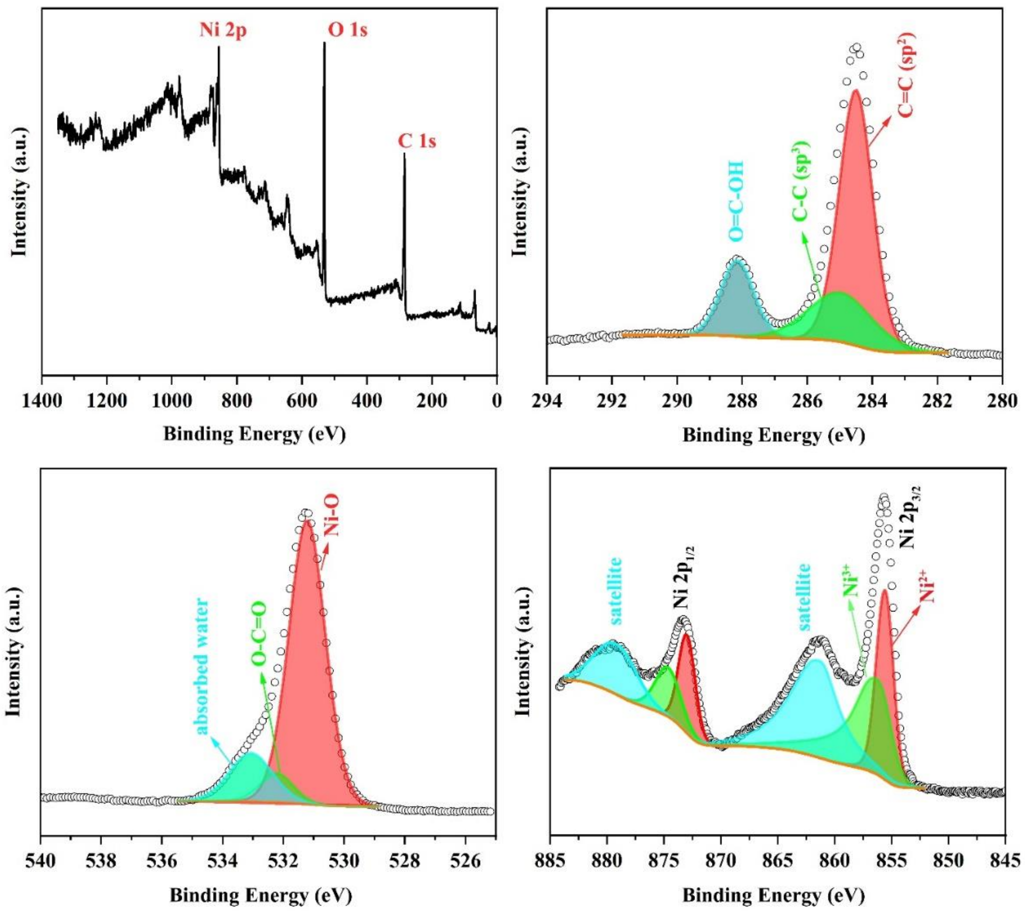

Figure S8. XPS survey, C 1s, O 1s, and Ni 2p spectra for Ni-BDC stripped off from NF.

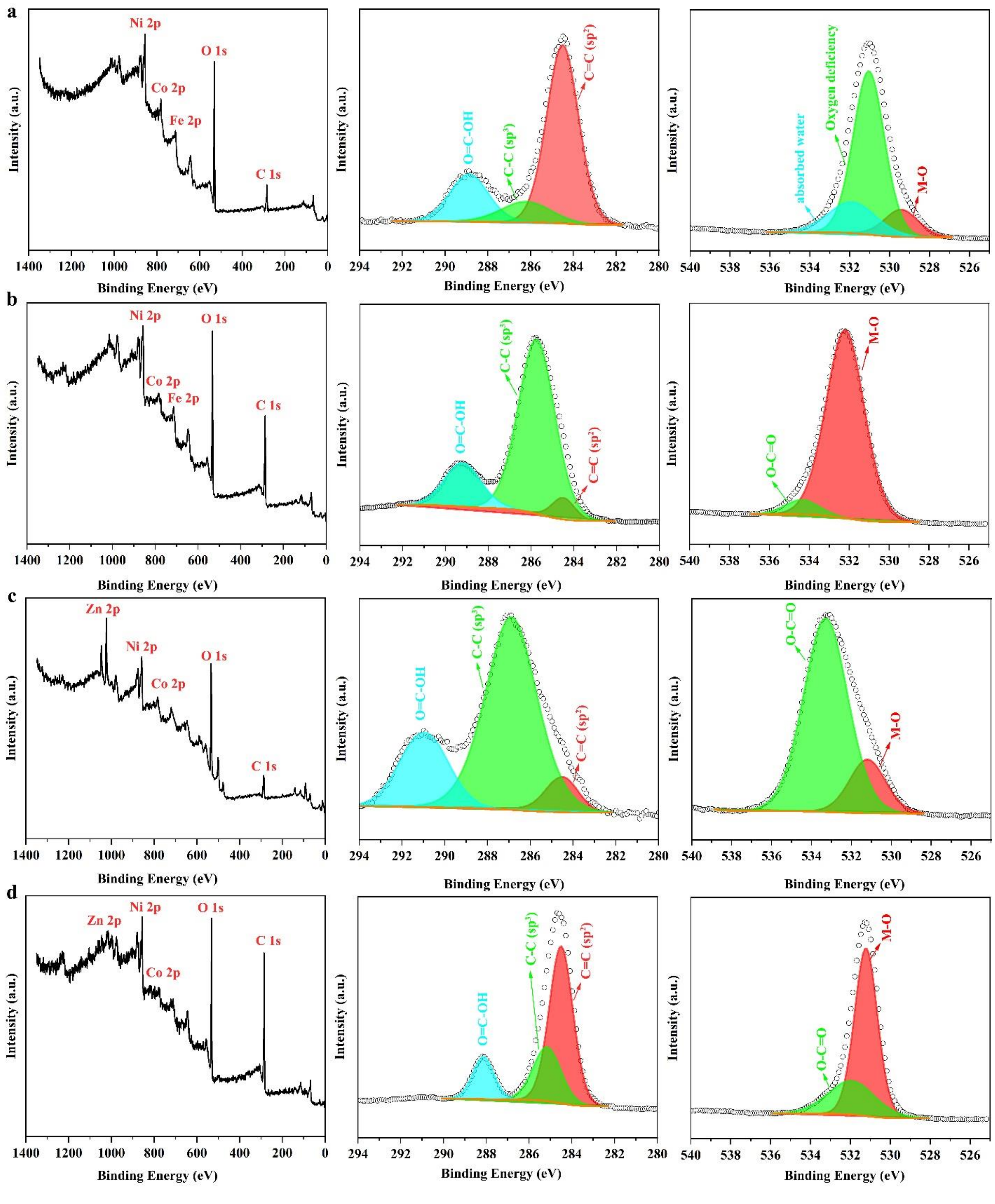

Figure S9. XPS survey, C 1s, and O 1s spectra for a) NCF, b) NCF/Ni-BDC, c) NCZ, and d) NCZ/Ni-BDC stripped off from NF.

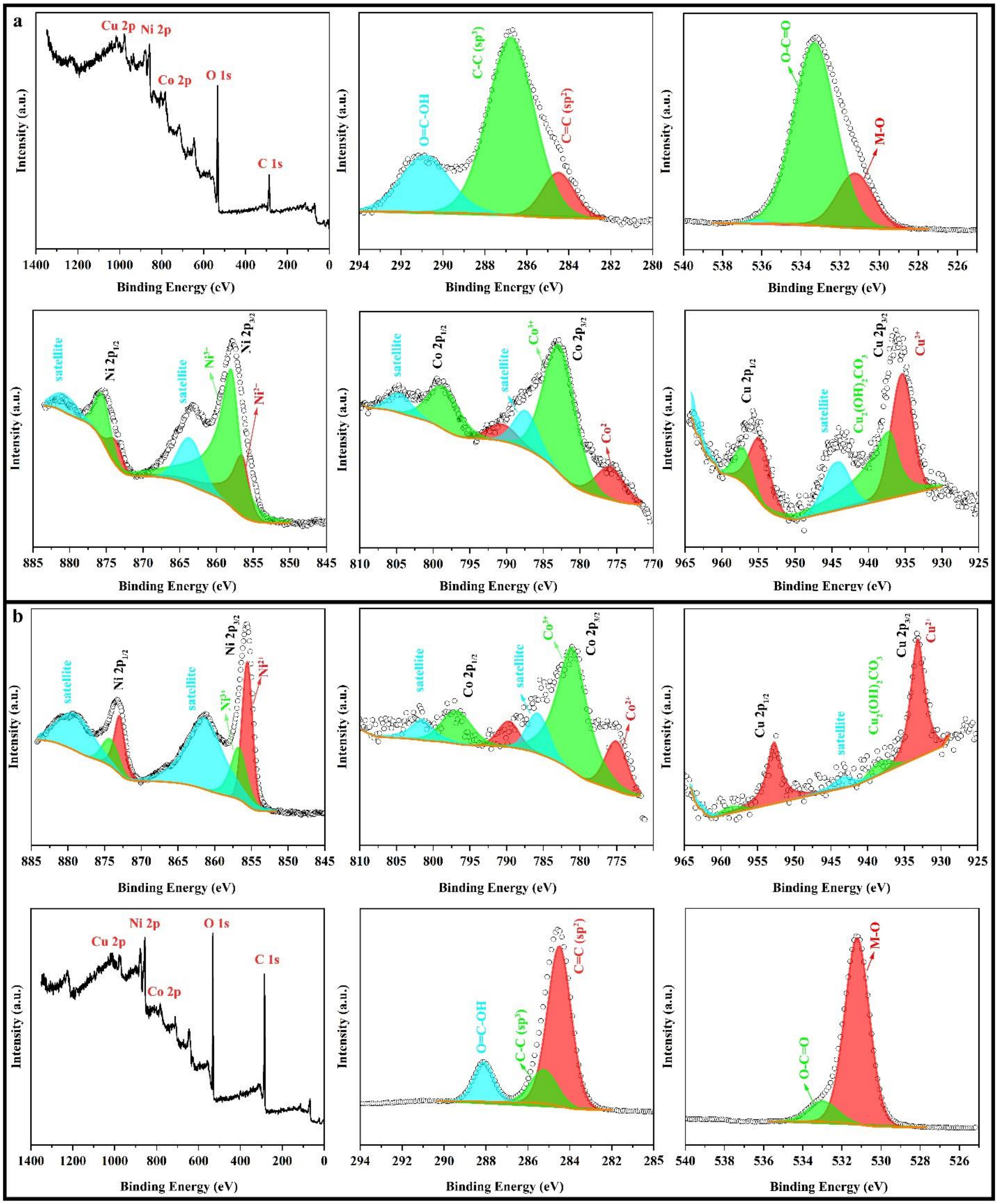

Figure S10. a) XPS survey, C 1s, O 1s, Ni 2p, Co 2p, and Cu 2p spectra for NCC and b) XPS Ni 2p, Co 2p, Cu 2p, survey, C 1s, and O 1s spectra for NCC/Ni-BDC stripped off from NF.

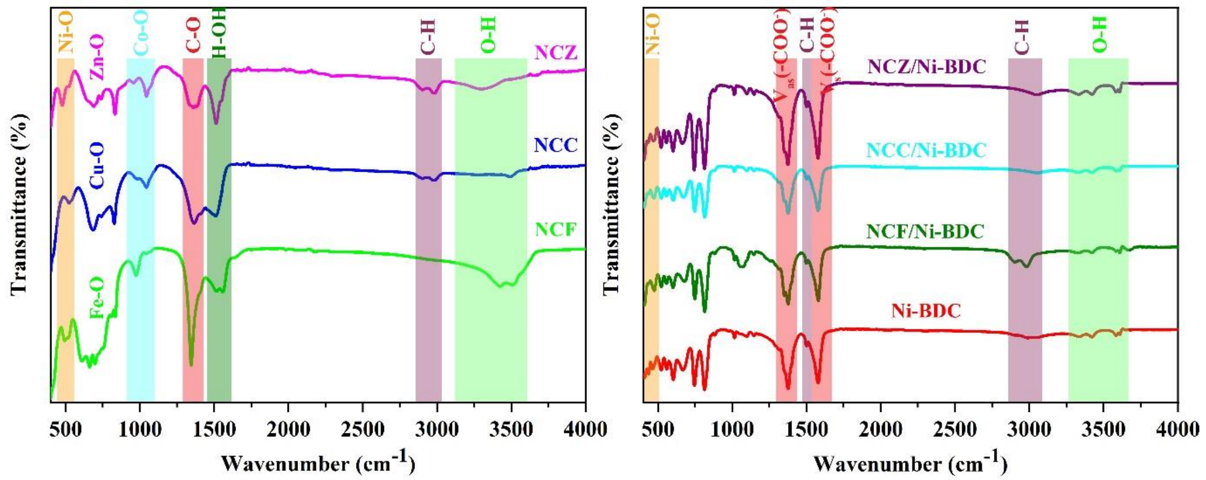

Figure S11. FT-IR spectra of ternary oxides and their corresponding nanocomposites stripped off from NF.

Table S2. The OER, HER, and overall water splitting performances of recent reports on MOF-derivative catalysts in terms of overpotential at 10 mA cm^{-2} (η^{10}) and 50 mA cm^{-2} (η^{50}) current densities.

Sample	η_{OER} (mV)	η_{HER} (mV)	Cell voltage (V)	Catalyst loading
Ni-BDC/NF	η^{10} = 436	-	-	12.8 mg cm^{-2}
Ni_3S_4/NF	η^{10} = 266	-	-	
Ni_3S_4/NF ‖ Ni_3S_4/NF [1]	-	-	1.638 @ 10 mA cm^{-2}	
NF@Ni/C-600	η^{10} = 265<br>η^{50} = 353	η^{10} = 37<br>η^{50} = 124	-<br>-	7.0 mg cm^{-2}
NF@Ni/C-600 ‖ NF@Ni/C-600 [2]	-	-	1.60 @ 10 mA cm^{-2}	
NiFe-LDH [3]	η^{50} = 270	-	-	-
NF@Fe_2-Ni_2P/C	η^{10} = 205	-	-	3.9 ± 0.3 mg cm^{-2}
NF@Fe_2-Ni/C	-	η^{10} = 33	-	
NF@Fe_2-Ni_2P/C ‖ NF@Fe_2-Ni_2P/C [4]	-	-	1.57 @ 100 mA cm^{-2}	
Fe-Ni@NC-CNT [5]	η^{10} = 274	η^{10} = 202	-	1.0 mg cm^{-2}

Ni@NC-800<br>Ni@NC-800NF ‖ Ni@NC-800NF [6]	η^{10} = 280<br>-	η^{10} = 205<br>-	-<br>1.60 @ 10 mA cm^{-2}	0.8 mg cm^{-2}
NiFe-MOF<br>NiFe-MOF ‖ NiFe-MOF [7]	η^{10} = 240<br>-	η^{10} = 134<br>-	-<br>1.55 @ 10 mA cm^{-2}	-
NFN-MOF/NF<br>NFN-MOF/NF ‖ NFN-MOF/NF [8]	η^{10} = 240<br>-	η^{10} = 87<br>-	-<br>1.56 @ 10 mA cm^{-2}	0.6 mg cm^{-2}
$(Ni_xFe_{1-x})_2P$<br>$(Ni_xFe_{1-x})_2P$ (1.0 mM) ‖ Ni_2P [9]	η^{10} = 166<br>-	η^{10} = 125<br>-	-<br>1.56 @ 10 mA cm^{-2}	1.0 mg cm^{-2}
Ru-doped NiFeP/NF<br>Ru-NiFeP/NF ‖ Ru-NiFeP/NF [10]	η^{10} = 179<br>-	η^{10} = 56<br>-	-<br>1.47 @ 10 mA cm^{-2}	10 mg cm^{-2}
Ni_3S_4<br>Ni_3S_4 ‖ Pt/C [11]	η^{10} = 257<br>η^{50} = 300<br>-	-<br>-<br>-	-<br>-<br>1.51 @ 10 mA cm^{-2}	11 mg cm^{-2}
$NiRu_{0.13}$-BDC [12]	-	η^{10} = 36	-	2.5 mg cm^{-2}
Zn–Ni–Co–S/NF<br>Zn–Ni–Co–P/NF<br>Zn–Ni–Co–S/NF ‖ Zn–Ni–Co–P/NF [13]	η^{50} = 190<br>-<br>-	-<br>η^{10} = 94<br>-	-<br>-<br>1.52 @ 10 mA cm^{-2}	-
Co-Fe oxide<br>Co-Fe oxide ‖ Co-Fe oxide [14]	η^{20} = 369<br>η^{100} = 406<br>-	η^{10} = 220<br>η^{100} = 290<br>-	-<br>-<br>1.92 @ 10 mA cm^{-2}	6.0 mg cm^{-2}
Ni-BDC<br>$Ni(OH)_2$	η^{10} = 358<br>η^{10} = 395	-<br>-	-<br>-	-

Ni-BDC/Ni(OH)$_2$ [15]	η^{10} = 320	-	-	
NiCo LDH@ZIF-67-V$_O$/NF [16]	η^{10} = 290	-	-	-

Table S3. The OER and HER performances of fabricated catalysts in this work in terms of overpotential at 10 mA cm^{-2} (η^{10}) and 50 mA cm^{-2} (η^{50}) current densities, charge transfer coefficient (α) for OER, and exchange current density (j^0) for HER.

**Sample**	**η^{10}_{OER} (mV)**	**η^{50}_{OER} (mV)**	**α**	**η^{10}_{HER} (mV)**	**η^{50}_{HER} (mV)**	**j^0 (mA cm^{-2})**
NF	380	-	0.23	314	435	0.017
RuO_2@NF	260	430	0.54	-	-	-
Pt/C@NF	-	-	-	61	214	1.08
Ni-BDC@NF	420	-	0.25	268	442	0.153
NCF@NF	160	350	1.49	231	383	0.316
NCC@NF	400	570	0.31	183	366	0.69
NCZ@NF	230	560	0.86	236	399	0.43
NCF/Ni-BDC@NF	120	450	1.68	215	373	0.382
NCC/Ni-BDC@NF	310	550	0.32	215	385	0.62
NCZ/Ni-BDC@NF	190	520	0.98	170	351	0.78

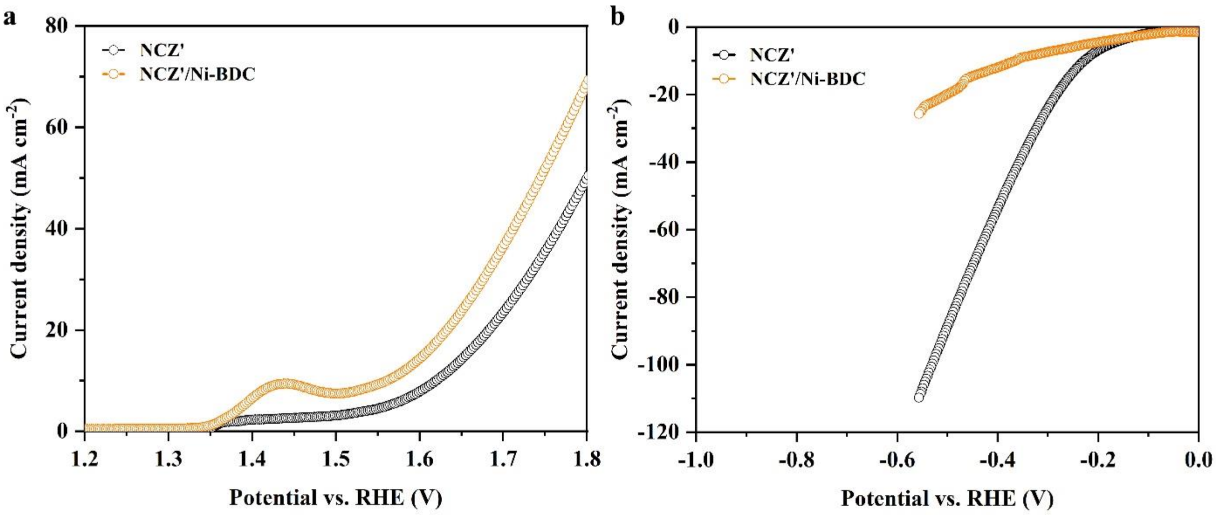

Figure S12. Electrochemical performance of NCZ’ and NCZ’/Ni-BDC, a) OER and b) HER.

Table S4. Estimated values obtained from the EIS simulations by the ZView software.

**Sample**	**R_e (Ω)**	**CPE1 ($mFsp^{-1}$)**	**R_p (Ω)**	**CPE2 ($mFsp^{-1}$)**	**R_{ct} (Ω)**
Ni-BDC	2.17	0.0018	51.08	0.0070	494.9
NCF	2.03	0.0017	41.06	0.0089	418.8
NCC	2.26	0.0022	49.5	0.0149	198.9
NCZ	1.76	0.0025	26.17	0.00012	45.7×10^4
NCF/Ni-BDC	2.02	0.0026	29.69	0.0151	116.3
NC*C*/Ni-BDC	1.96	0.0021	52.96	0.0133	167
NCZ/Ni-BDC	1.97	0.002	17.31	0.0159	126.9
Ni-BDC (-) || Ni-BDC (+)	5.17	0.0019	2607	0.00083	37.8×10^9
NCC (-) || NCF (+)	6.38	0.0021	3216	0.0012	2818
NCZ/Ni-BDC (-) || NCF/Ni-BDC (+)	4.70	0.025	27.9	0.023	423

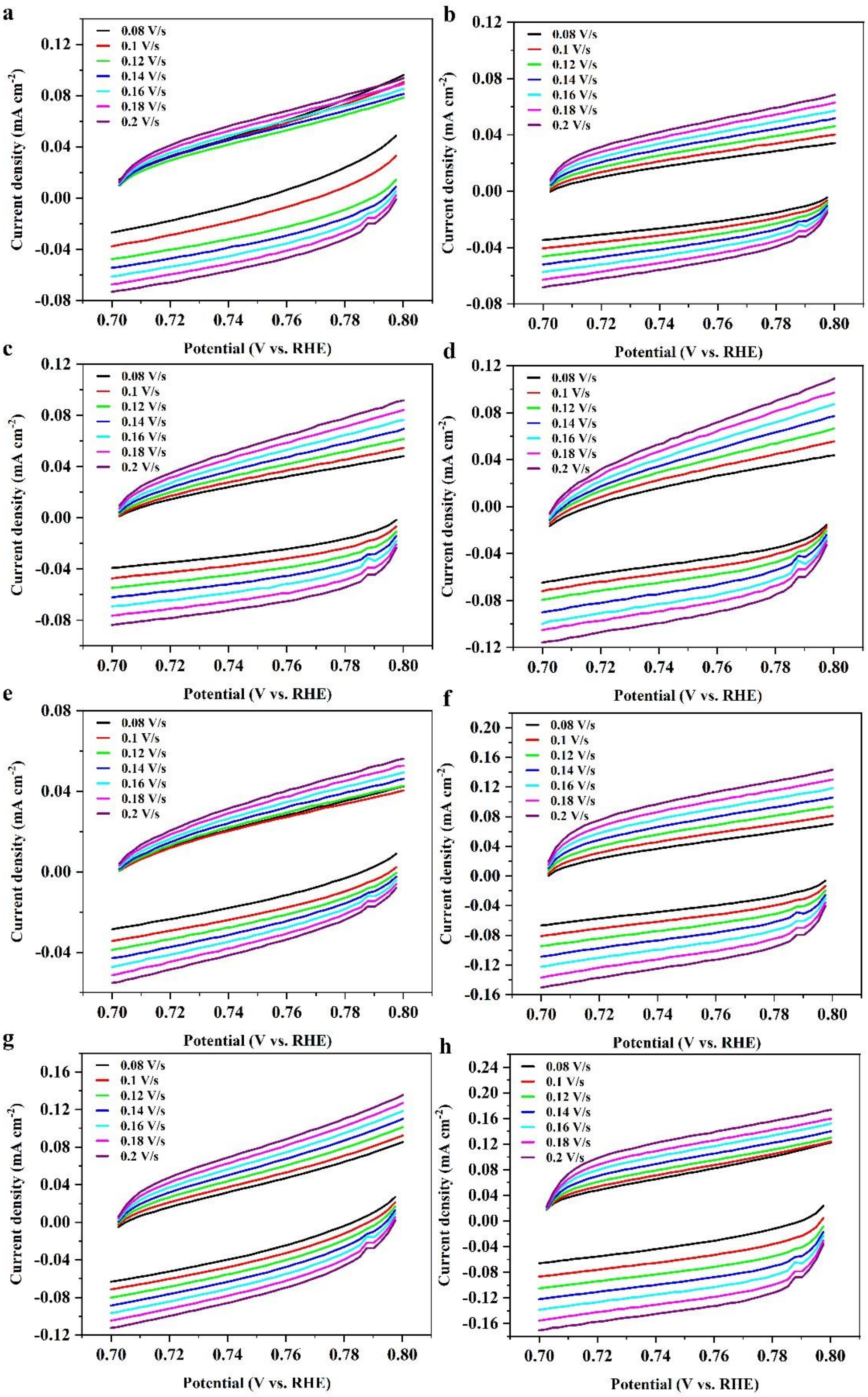

Figure S13. Typical cyclic voltammetry (CV) curves obtained at different scan rates (0.08–0.2 V s^{-1}) within the potential window 0.7–0.8 V vs RHE for a) blank NF, b) Ni-BDC, c) NCF, d) NCC, e) NCZ, f) NCF/Ni-BDC, g) NCC/Ni-BDC, and h) NCZ/Ni-BDC.

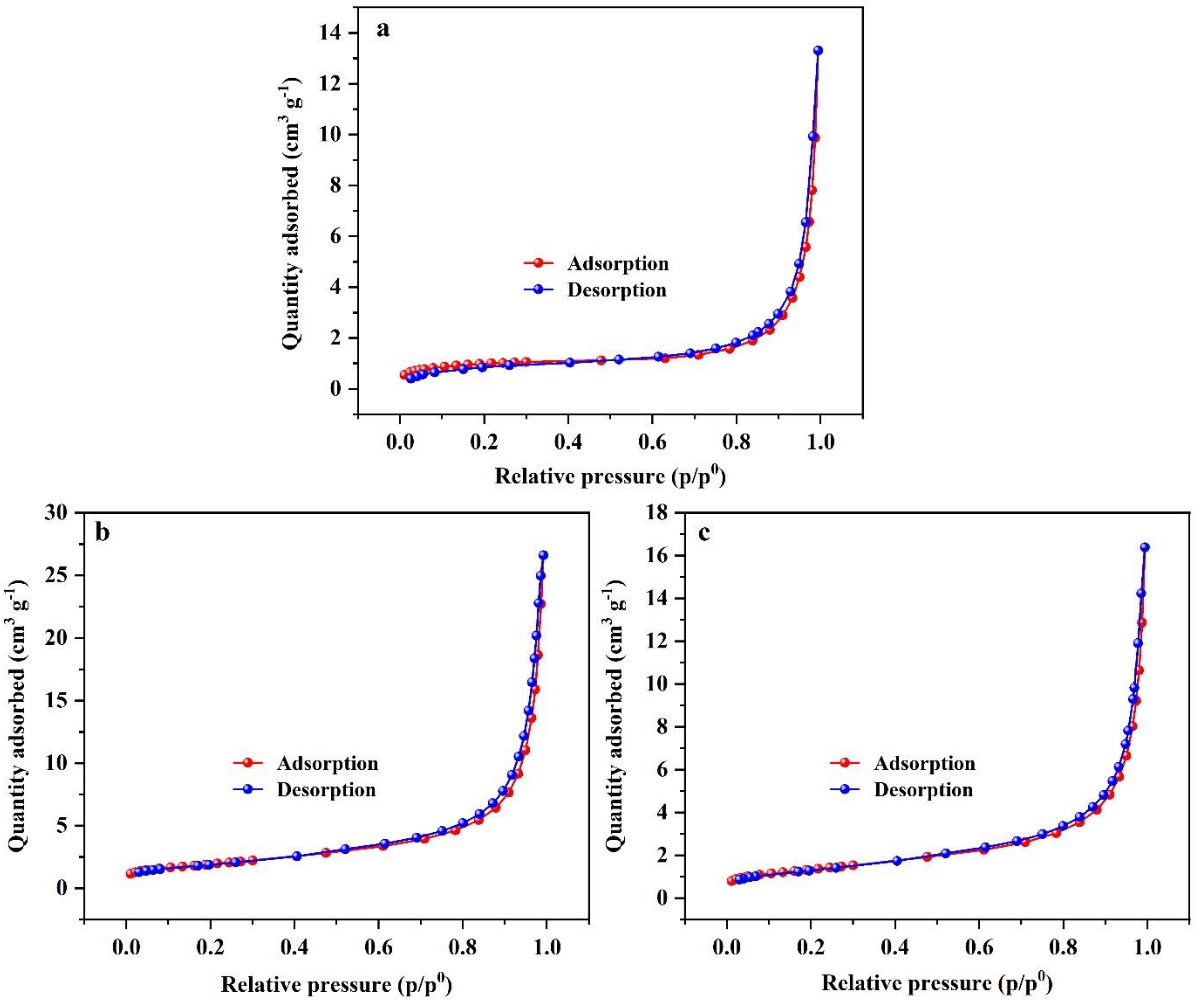

Figure S14. N_2 adsorption-desorption isotherms of a) Ni-BDC, b) NCF/Ni-BDC, and c) NCZ/Ni-BDC.

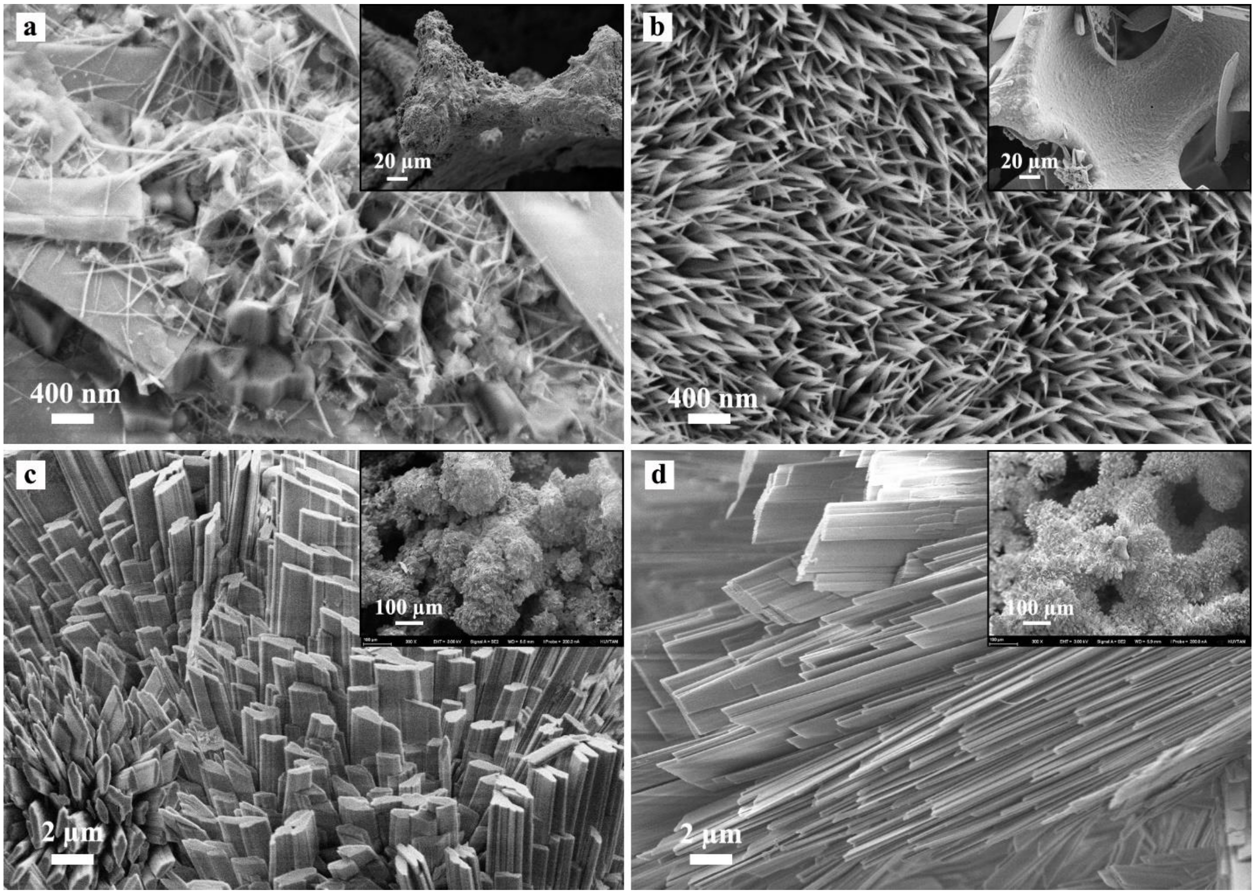

Figure S15. SEM images of a) NCF and c) NCF/Ni-BDC after OER tests, and b) NCC and d) NCZ/Ni-BDC after HER tests.

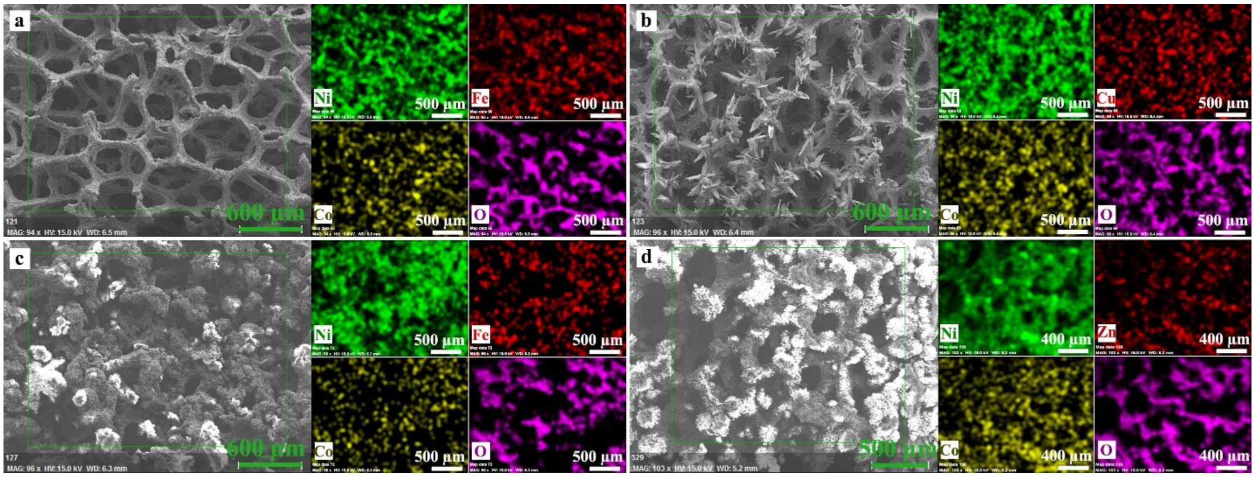

Figure S16. SEM/EDS elemental mappings of a) NCF and c) NCF/Ni-BDC after OER tests, and b) NCC and d) NCZ/Ni-BDC after HER tests.

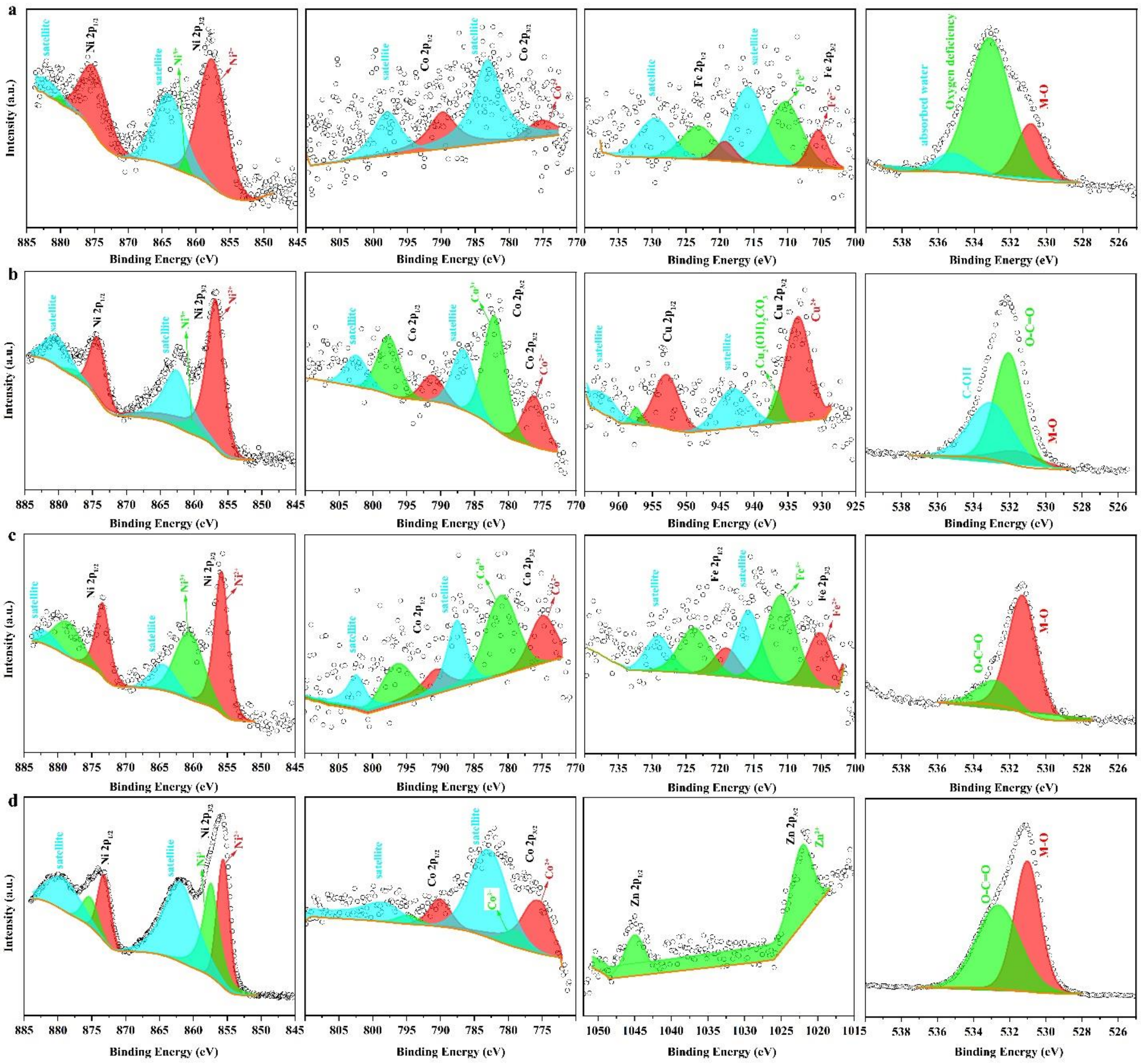

Figure S17. XPS spectra after electrochemical OER and HER measurements. a and c) Ni 2p, Co 2p, Fe 2p, and O 1s for NCF and NCF/Ni-BDC after OER tests and b and d) Ni 2p, Co 2p, Cu 2p, and O 1s for NCC and NCZ/Ni-BDC after HER tests.

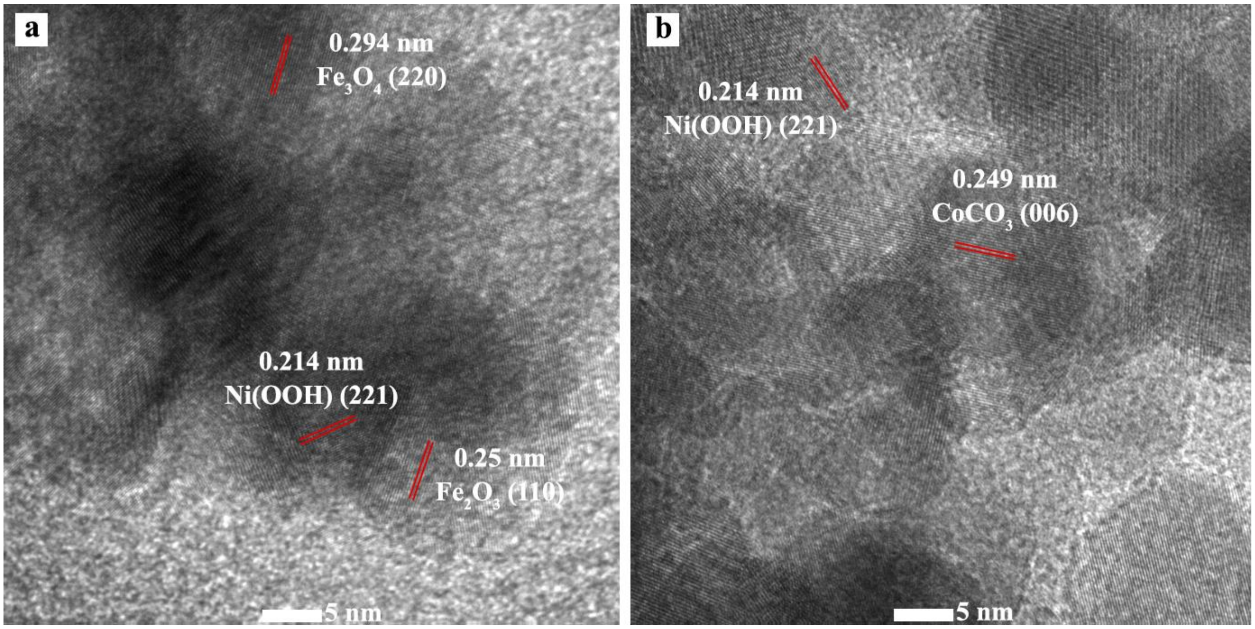

Figure S18. Post-electrolysis HR-TEM photographs. a) NCF/Ni-BDC after the OER durability test and b) NCZ/Ni-BDC after the HER stability test.

Table S5. ICP-MS analysis on dissolved elements during OER stability for NCF/Ni-BDC and HER stability for NCZ/Ni-BDC.

Sample	Fe [Conc. (ppb)]	Co [Conc. (ppb)]	Zn [Conc. (ppb)]	Ni [Conc. (ppb)]
NCF/Ni-BDC	9.55	0.14	-	-
NCZ/Ni-BDC	17.81	3.21	40.82	-

References:

1. Li, N.; Ai, L.; Jiang, J.; Liu, S., Spinel-type oxygen-incorporated Ni3+ self-doped Ni3S4 ultrathin nanosheets for highly efficient and stable oxygen evolution electrocatalysis. *Journal of colloid and interface science* **2020,** *564*, 418-427.
2. Sun, H.; Lian, Y.; Yang, C.; Xiong, L.; Qi, P.; Mu, Q.; Zhao, X.; Guo, J.; Deng, Z.; Peng, Y., A hierarchical nickel–carbon structure templated by metal–organic frameworks for efficient overall water splitting. *Energy & Environmental Science* **2018,** *11* (9), 2363-2371.
3. Zhou, Y.-N.; Yu, W.-L.; Cao, Y.-N.; Zhao, J.; Dong, B.; Ma, Y.; Wang, F.-L.; Fan, R.-Y.; Zhou, Y.-L.; Chai, Y.-M., S-doped nickel-iron hydroxides synthesized by room-temperature electrochemical activation for efficient oxygen evolution. *Applied Catalysis B: Environmental* **2021,** *292*, 120150.
4. Sun, H.; Min, Y.; Yang, W.; Lian, Y.; Lin, L.; Feng, K.; Deng, Z.; Chen, M.; Zhong, J.; Xu, L., Morphological and electronic tuning of Ni2P through iron doping toward highly efficient water splitting. *ACS Catalysis* **2019,** *9* (10), 8882-8892.

5. Zhao, X.; Pachfule, P.; Li, S.; Simke, J. R. J.; Schmidt, J.; Thomas, A., Bifunctional Electrocatalysts for Overall Water Splitting from an Iron/Nickel- Based Bimetallic Metal–Organic Framework/Dicyandiamide Composite. *Angewandte Chemie* **2018,** *130* (29), 9059-9064.
6. Xu, Y.; Tu, W.; Zhang, B.; Yin, S.; Huang, Y.; Kraft, M.; Xu, R., Nickel nanoparticles encapsulated in few- layer nitrogen- doped graphene derived from metal–organic frameworks as efficient bifunctional electrocatalysts for overall water splitting. *Advanced Materials* **2017,** *29* (11), 1605957.
7. Duan, J.; Chen, S.; Zhao, C., Ultrathin metal-organic framework array for efficient electrocatalytic water splitting. *Nature communications* **2017,** *8* (1), 1-7.
8. Senthil Raja, D.; Chuah, X. F.; Lu, S. Y., In situ grown bimetallic MOF- based composite as highly efficient bifunctional electrocatalyst for overall water splitting with ultrastability at high current densities. *Advanced Energy Materials* **2018,** *8* (23), 1801065.
9. Sun, S.; Zhou, X.; Cong, B.; Hong, W.; Chen, G., Tailoring the d-Band Centers Endows (Ni x Fe1–x) 2P Nanosheets with Efficient Oxygen Evolution Catalysis. *ACS Catalysis* **2020,** *10* (16), 9086-9097.
10. Lin, Y.; Zhang, M.; Zhao, L.; Wang, L.; Cao, D.; Gong, Y., Ru doped bimetallic phosphide derived from 2D metal organic framework as active and robust electrocatalyst for water splitting. *Applied Surface Science* **2021,** *536*, 147952.
11. Wan, K.; Luo, J.; Zhou, C.; Zhang, T.; Arbiol, J.; Lu, X.; Mao, B. W.; Zhang, X.; Fransaer, J., Hierarchical porous Ni3S4 with enriched high- valence Ni sites as a robust electrocatalyst for efficient oxygen evolution reaction. *Advanced Functional Materials* **2019,** *29* (18), 1900315.
12. Sun, Y.; Xue, Z.; Liu, Q.; Jia, Y.; Li, Y.; Liu, K.; Lin, Y.; Liu, M.; Li, G.; Su, C.-Y., Modulating electronic structure of metal-organic frameworks by introducing atomically dispersed Ru for efficient hydrogen evolution. *Nature communications* **2021,** *12* (1), 1-8.
13. Ding, Y.; Du, X.; Zhang, X., Controlled synthesis and high performance of Zn–Ni–Co–M (M= O, S, P and Se) nanoneedle arrays as an advanced electrode for overall water splitting. *Applied Surface Science* **2021,** *543*, 148818.
14. Adamson, W.; Bo, X.; Li, Y.; Suryanto, B. H.; Chen, X.; Zhao, C., Co-Fe binary metal oxide electrocatalyst with synergistic interface structures for efficient overall water splitting. *Catalysis Today* **2020,** *351*, 44-49.
15. Zhu, D.; Liu, J.; Wang, L.; Du, Y.; Zheng, Y.; Davey, K.; Qiao, S.-Z., A 2D metal–organic framework/Ni (OH) 2 heterostructure for an enhanced oxygen evolution reaction. *Nanoscale* **2019,** *11* (8), 3599-3605.
16. Chen, W.; Zhang, Y.; Huang, R.; Zhou, Y.; Wu, Y.; Hu, Y.; Ostrikov, K. K., Ni–Co hydroxide nanosheets on plasma-reduced Co-based metal–organic nanocages for electrocatalytic water oxidation. *Journal of Materials Chemistry A* **2019,** *7* (9), 4950-4959.
